# Chinese Patent Medicine as Adjuvant for Mild-to-Moderate Active Ulcerative Colitis: A Network Meta-Analysis of Randomized Controlled Trials

**DOI:** 10.1155/2021/1075886

**Published:** 2021-08-26

**Authors:** Yu-Xin Sun, Guo-Yan Yang, Diana Karamacoska, Xiao Wang, Yuan-Xi Li, Wen-Bin Hou, You-You Zheng, Jian-Ping Liu, Zhao-Lan Liu

**Affiliations:** ^1^Centre for Evidence-Based Chinese Medicine, Beijing University of Chinese Medicine, Beijing 100029, China; ^2^NICM Health Research Institute, Western Sydney University, Penrith, NSW 2751, Australia; ^3^College of Statistics and Data Science, Faculty of Science, Beijing University of Technology, Beijing 100124, China

## Abstract

**Objective:**

To evaluate the effectiveness and safety of Chinese patent medicine for mild-to-moderate active ulcerative colitis (UC) using network meta-analysis (NMA).

**Methods:**

We systematically searched PubMed, Cochrane library, Embase, Sino-Med, China National Knowledge Infrastructure (CNKI), Wanfang, and Chinese Scientific Journal Database (VIP) databases to October, 2020. We included randomized controlled trials (RCTs) on Chinese patent medicine for mild-to-moderate active UC. The main analysis was complemented by network subanalyses and standard pairwise comparisons. Statistical heterogeneity, inconsistencies, and ranking probability were also evaluated.

**Results:**

The databases search identified 3222 citations, of which 33 RCTs involving 2971 patients met the inclusion criteria. A total of 15 Chinese patent medicines were analyzed. The overall quality of the included studies was low. Pairwise meta-analysis showed that Chinese patent medicine was superior to Mesalazine in improving disappearances of clinical symptoms, recurrence rate, and Mayo score. Based on decreases in adverse events, results from NMA showed that Xilei powder plus Mesalazine was more effective than other drugs. Other NMA results indicated that Danshen freeze-dried powder plus Mesalazine (RR: 0.13; 95% CI, 0.02–0.78) and Kangfuxin lotion plus Mesalazine (RR: 0.24; 95% CI, 0.07–0.57) were superior to Mesalazine in decreasing recurrence rate. Another NMA result indicated that Kangfuxin lotion plus Mesalazine (RR: 0.00; 95% CI, 0.00–0.02) and Zhi Kang capsule plus Mesalazine (RR: 0.00; 95% CI, 0.00–0.02) were superior to Mesalazine in increasing the disappearance of tenesmus.

**Conclusion:**

In the probability sorting, Xilei powder combined with Mesalazine ranked first for having the fewest adverse events, Maintaining Intestines Antidiarrheal Pills combined with Mesalazine ranked first for having the lowest recurrence rate, Xilei powder combined with Mesalazine ranked first for improving disappearance rate of mucopurulent bloody stool/abdominal pain, and Kangfuxin lotion combined with Mesalazine ranked first for improving the disappearance rate of diarrhea/tenesmus. However, there is a lack of direct comparisons among Chinese patent medicines for UC. More multiarm RCTs are needed in the future to provide direct comparative evidence.

## 1. Introduction

Ulcerative colitis (UC) is a chronic inflammatory bowel disease characterized by change of stool excretion habits, diarrhea, and hematochezia which occurs in the mucosa and submucosa from the rectum to the colon [1]. The pathogenesis of UC is complex, which is closely related to genetic susceptibility, lifestyle, intestinal flora imbalance, and immune disorder [2–5]. The prevalence rate of UC in China is 11.6/100000, which is higher than that in Japan and South Korea, and it ranks the top among Asian countries. The earliest case report of UC in China was reported in 1950s by China Union Hospital [6]. The incidence rate of this disease increased dramatically. The study showed that 10218 cases of UC were reported in 1560 papers published in the 20 years from 1981 to 2000 [7]. The number of diagnostic reports in ten years was about 2.99 times that of the first ten years.

The current pharmaceutical treatments of UC include anti-inflammatory drugs, immunosuppressant drugs, biologics, and other over-the-counter medications. However, some drugs have serious side effects. For example, sulfasalazine can cause severe drug eruption, drug-induced hypersensitivity syndrome, agranulocytosis followed by typhoid infection, and acute pancreatitis in patients [8–11]. In addition, some patients fail to respond to first-line drugs such as salicylic acid preparations and immunosuppressant drugs or become hormone resistant or dependent. As a result, in China, many patients tend to use traditional Chinese medicine (TCM), including Chinese patent medicine in treating UC [12].

Chinese patent medicine is a kind of ready-to-use form of TCM products, which is prepared from raw Chinese herbal medicines based on the prescription and preparation process under the guidance of Yin and Yang, five elements, meridian and collateral, and other theories of TCM. Chinese patent medicines have specific names, usages and dosages, specifications, specific quality standards, and inspection methods, as well as clear description of contraindications and precautions. Several randomized controlled trials (RCTs) have demonstrated the potential effect and safety of these Chinese patent medicines in the treatment of mild-to-moderate active UC. Although previous two network meta-analyses focused on the effectiveness of different TCM injections combined with western medicine in the treatment of mild-to-moderate UC [13, 14], the relative effectiveness and safety between these treatment options remain unclear due to the lack of head-to-head comparisons.

The objective of this systematic review (SR) and network meta-analysis (NMA) of RCTs was to assess the relative effectiveness and safety of Chinese patent medicine in patients with mild-to-moderate active UC.

## 2. Methods

This NMA was conducted under the guidance of the Cochrane Handbook for Systematic Reviews of Interventions [15], in particular Chapter 11: undertaking network meta-analyses, and reported in accordance with the Preferred Reporting Items for Systematic Reviews and Meta-Analyses (PRISMA) extension statement for reporting of SR and NMA [16]. The protocol of this study has been registered in the International Prospective Register for Systematic Reviews (PROSPERO), CRD42020213867 (https://www.crd.york.ac.uk/prospero/).

### 2.1. Identification and Selection of Studies

We searched relevant RCTs from PubMed, Cochrane library, Embase, SinoMed, China National Knowledge Infrastructure (CNKI), Wanfang, and VIP databases to identify studies from their inception up to October, 2020. The searching words included “ulcerative colitis,” “UC,” “Chinese patent medicine,” “Chinese patent drug,” “Chinese proprietary medicine,” “randomized controlled trial,” “randomly,” “xileisan,” “bawei xileisan,” “yunnan baiyao,” “fufangkushen colon-coated capsule,” and “zhikang capsule”. For each database, we established a corresponding retrieval strategy with Boolean formula. PubMed search strategy was detailed in Table 1. Two reviewers independently screened citations against the following predefined selection criteria.

### 2.2. Population

We included participants diagnosed as mild-to-moderate active UC. There is no limitation in age, sex, nation, ethnicity, and disease stage. Children and pregnant women were excluded. Patients were excluded if they had other intestinal diseases including bacterial dysentery, amoeba colitis, schistosomiasis, intestinal tuberculosis, Crohn's disease, and reflex enteritis.

### 2.3. Interventions

Chinese patent medicines with the approval batch number beginning with “Z,” approved by National Pharmaceutical Regulatory Body in China, used alone or in combination with Mesalazine (i.e., 5-aminosalicylic acid) or placebo, are eligible. No limitation was applied on drug dosage, drug formulations, and route of administration. The minimum treatment duration was at least 14 days.

### 2.4. Comparators

Other Chinese patent medicines, Mesalazine (i.e., 5-aminosalicylic acid (5-ASA), drugs recommended by international authorized clinical guidelines [17]), or placebo were eligible for inclusion.

### 2.5. Outcomes

Primary outcomes are the disappearance of symptoms including abdominal pain, diarrhea and bloody purulent stool, and Mayo score. Secondary outcomes include adverse events and recurrence rate measured in the follow-up visit from the end of treatment to two years.

### 2.6. Study Design

We included parallel RCTs. Conference papers were excluded if full papers were not available.

### 2.7. Study Selection

Two reviewers (YXS and XW) independently screened the study titles and abstracts, identifying studies that met the inclusion criteria for full-text evaluation. In studies with at least three arms, any arm not relevant to our analysis was excluded. We resolved any disagreement about the study selection through discussion, with a third author (ZLL) involved when necessary.

### 2.8. Data Collection Process and Data Items

The two reviewers (YXS and XW) independently extracted the information and data including study ID, publication year, interventions, outcome measures, and funding information. Any disagreement about data extraction was resolved by discussion with involvement of a third author (WBH) when necessary.

### 2.9. Risk of Bias Assessment

Two reviewers (YXL, YYZ) independently assessed the risk of bias for each included trial using the Cochrane's Risk of Bias tool. We resolved any disagreements by consensus or by consulting a third review author (ZLL).

### 2.10. Statistical Analysis

#### 2.10.1. Pairwise Meta-Analyses

Dichotomous data and continuous data were analyzed by Cochrane's Review Manager software (version 5.3). *I*^2^ values were used to evaluate the statistical heterogeneity between the included studies. When there are no or low heterogeneity between studies (*I*^2^ < 25%), the fixed effect model was used for pooling data. If there is substantial heterogeneity (25% < *I*^2^ < 95%) and clinical heterogeneity was deemed acceptable, we used random-effects model to conduct the meta-analysis. When the statistical heterogeneity is particularly large (*I*^2^ > 95%) or clinical heterogeneity is particularly significant, we did not pool the quantitative data. We used relative ratio (RR) with 95% confidence interval (CI) for dichotomous variables and for mean differences (MD) with 95% CI for continuous variables.

#### 2.10.2. Network Meta-Analyses

Aggregate Data Drug Information System (ADDIS software 1.16.5) and STATA 16 software were used to perform the Bayesian NMA to compare direct and indirect evidence. The Markov Chain Monte Carlo (MCMC) was used to simulate the data, and chains and different iterations (number of annealing times) were set [18]. The test models used in this study were the random effects and consistency models. The degree of convergence of the model was evaluated by the Brooks-Gelman-Rubin method with the potential scale reduction factor (PSRF) as evaluation indicator. PSRF values close to 1 indicate better convergence effect of the model, and generally PSRF values less than 1.05 are acceptable [19]. RR, MD, and 95% CI were used to summarize data. We examined the consistency of NMA by using the node-splitting analysis method. For a closed loop of three treatments, the inconsistency between direct and indirect evidence was directly assessed. The probability of each intervention being the best for each outcome was calculated and reported in the form of rank grams. The rank of each treatment is shown on the histogram, which indicates the probability of being ranked in that position. Lower ranks indicate a better effect. Rank 1 is best; Rank *N* is worst.

### 2.11. Consistent Assessment and Publication Bias

Due to the lack of head-to-head comparisons, no node-splitting method was formed and 95% CI of inconsistence factors could not be generated, so the node-splitting model was not used for consistency test. We did not perform funnel plots to detect potential publication bias because there were no more than 10 included RCTs in each meta-analysis.

## 3. Results

### 3.1. Study Selection

**Figure 1:**
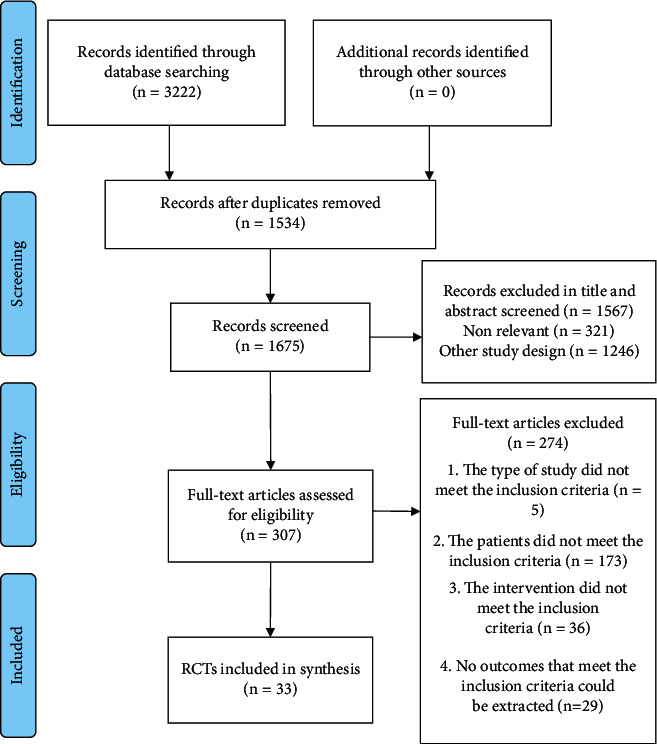
PRISMA flow program for study selection.

We identified 3222 potential articles in the initial search. A total of 1534 duplicate articles were excluded. We screened the remaining abstracts and 307 full-text articles for potentially eligible studies. Finally, 33 RCTs were included in this review (Figure 1).

### 3.2. Characteristics of the Selected Literature

The characteristics of the included studies are summarized in Table 2 [20–52]. A total of 33 studies, published between 2007 and 2019, involving 2862 patients with active mild-to-moderate UC, were included in this review. Among them, 55% (1209/2169) were male patients. All included studies were conducted in China. A total of 18 interventions were identified in included studies, including 3 RCTs that used Chinese patent medicine alone and 15 Chinese patent medicines used in combination with Mesalazine or placebo.

### 3.3. Methodological Quality

**Figure 2:**
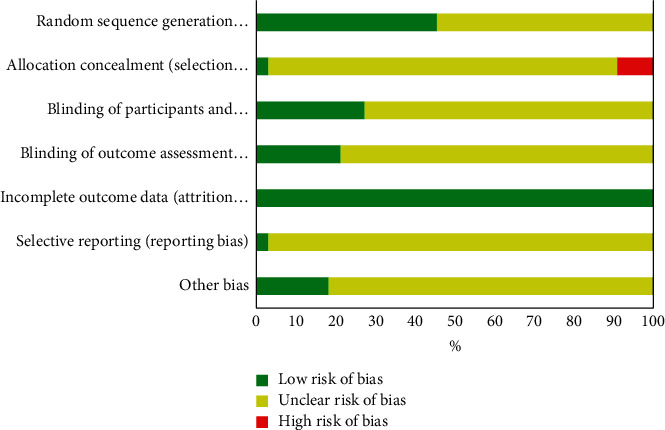
The risk of bias of all the final included RCTs.

The risk of bias assessment is shown in Figure 2 and Table 3. The overall methodological quality of included studies was poor. Only one trial had high methodology quality with a low risk of bias for each item [20]. The method of random sequence generation was not explained in 42.4% (14/33) trials. Blinding was used in three trials [20–22]. None of the trials used blinding in outcome assessment. One trial registered the protocol [20]. 18.2% (6/33) trials reported funding information. No sample size estimates were reported in all trials. A portion of RCTs did not specify the inclusion and exclusion criteria for patients.

### 3.4. Results of Individual Studies and Synthesis of Results

#### 3.4.1. Results of Pairwise Meta-Analysis

The detailed results were provided in Table 4 and Supplementary S1. Kangfuxin lotion plus Mesalazine and Danshen freeze-dried powder plus Mesalazine were significantly superior to Mesalazine alone in reducing recurrence rate. Kangfuxin lotion plus Mesalazine was statistically superior to Mesalazine alone in disappearance of mucopurulent bloody stool. Combined treatments including enteric-coated Hudi capsules plus Mesalazine and enteric-coated Hudi capsules plus placebo were statistically superior to Mesalazine plus placebo in disappearance of mucopurulent bloody stool. There were statistically significant differences between combined treatments including Xilei powder plus Mesalazine, Kangfuxin lotion plus Mesalazine, and Bupi Yichang pill plus Mesalazine and Mesalazine alone in disappearance of abdominal pain, favoring the combined treatments. Statistically significant differences were also seen between combined treatments including Kangfuxin lotion plus Mesalazine and Bupi Yichang pill plus Mesalazine and Mesalazine alone in disappearance of diarrhea, favoring the combined treatments. Chinese patent medicines combined with Mesalazine also showed better effects than Mesalazine alone in disappearance of tenesmus. Danshen Injection plus Mesalazine was statistically superior to Mesalazine in Mayo score. No statistically significant differences in other outcomes were found.

#### 3.4.2. Results of the Network Meta-Analysis

**Figure 3:**
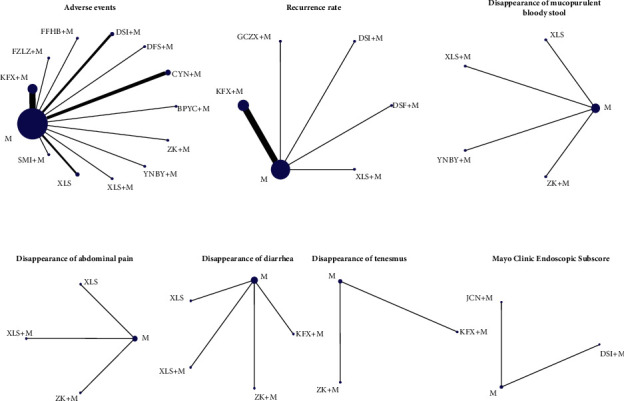
Network plot comparing the adverse events, recurrence rate, disappearance of mucopurulent bloody stool/abdominal pain/diarrhea/tenesmus, and Mayo score. Each node represents a treatment, connections between nodes represent direct comparisons, and node sizes and the thickness of connections vary according to the number of studies involved in a comparison.

The networks for comparison established in this study are presented in Figure 3. The detailed results are summarized in Table 5. All the PSFR value of operation time ranged from 1.00 to 1.05, indicating complete convergence, good iterative effects, and stable results of the model. In terms of adverse events, Xilei Powder was safer than the remaining 12 interventions, including Aconitum Lizhong pill plus Mesalazine, Xilei powder plus Mesalazine, Bupi Yichang pill plus Mesalazine, Zhikang capsule plus Mesalazine, ChangYanNing capsule plus Mesalazine, Mesalazine, Shengmai injection plus Mesalazine, Kangfuxin lotion plus Mesalazine, compound Huangbai liquid plus Mesalazine, Yunnan white drug-powder plus Mesalazine, Danshen freeze-dried powder plus Mesalazine, and Danshen injection plus Mesalazine. Mesalazine-combined treatments, Kangfuxin lotion plus Mesalazine and Danshen freeze-dried powder plus Mesalazine, were superior to Mesalazine alone in reducing recurrence rate, while for disappearance of tenesmus, the combined treatment Kangfuxin lotion plus Mesalazine was less effective than Mesalazine and Zhikang capsule plus Mesalazine. No statistically significant difference was found in other outcomes.

### 3.5. Rank Probabilities

**Figure 4:**
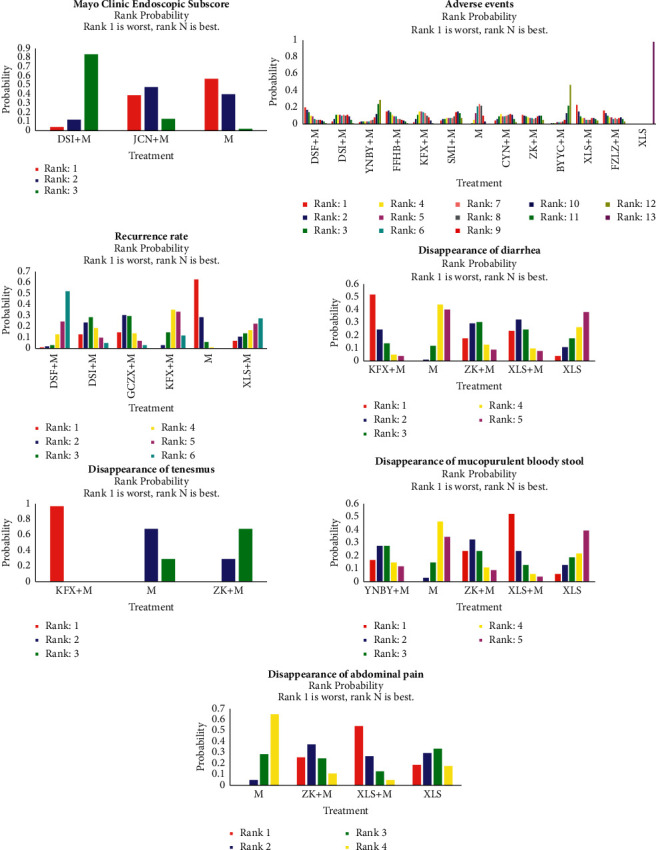
Rank probability for the adverse events, recurrence rate, disappearance of mucopurulent bloody stool/abdominal pain/diarrhea/tenesmus, and Mayo score.

The probability rankings are shown in Table 6 and Figure 4. The rank of each treatment is shown on the histogram, which indicates the probability of being ranked in that position. Lower rank indicates a better effect. Rank 1 is best, and Rank *N* is worst.

#### 3.5.1. Adverse Events

The cumulative probability of having the fewest adverse events was Xilei powder plus Mesalazine (23%), followed by Danshen freeze-dried powder plus Mesalazine, compound Huangbai liquid plus Mesalazine, Kangfuxin lotion plus Mesalazine, Mesalazine, Shengmai injection plus Mesalazine, Yunnan white drug-powder plus Mesalazine, Bupi Yichang pill plus Mesalazine, and Xilei powder alone.

#### 3.5.2. Recurrence Rate

The cumulative probability of having the lowest recurrence rate was Mesalazine (64%), followed by Maintaining Intestines Antidiarrheal Pills plus Mesalazine, Danshen injection plus Mesalazine, Kangfuxin lotion plus Mesalazine, Danshen freeze-dried powder plus Mesalazine, and Zhikang capsule plus Mesalazine.

#### 3.5.3. Disappearance of Mucopurulent Bloody Stool

The cumulative probability of being the most efficacious treatment in improving disappearance of mucopurulent bloody stool was Xilei powder plus Mesalazine (53%), followed by Zhikang capsule plus Mesalazine, Yunnan white drug-powder plus Mesalazine, Mesalazine alone, and Xilei powder alone.

#### 3.5.4. Disappearance of Abdominal Pain

The cumulative probability of being the most efficacious treatment in improving disappearance of abdominal pain was Xilei powder plus Mesalazine (55%), followed by Zhikang capsule plus Mesalazine, Xilei powder alone, and Mesalazine alone.

#### 3.5.5. Disappearance of Diarrhea

The cumulative probability of being the most efficacious treatment in improving diarrhea was Kangfuxin lotion plus Mesalazine (53%), followed by Xilei powder plus Mesalazine, Zhikang capsule plus Mesalazine, Mesalazine alone, and Xilei powder alone.

#### 3.5.6. Disappearance of Tenesmus

The cumulative probability of being the most efficacious treatment in improving tenesmus was Kangfuxin lotion plus Mesalazine (100%), followed by Mesalazine, and Zhikang capsule plus Mesalazine.

#### 3.5.7. Mayo Score

The cumulative probability of being the most efficacious treatment in improving Mayo score was Mesalazine (57%), followed by JieChangNing Capsule plus Mesalazine and Danshen injection plus Mesalazine.

## 4. Discussion

### 4.1. Main Finding

To our knowledge, this is the first SR and NMA evaluating the relative effectiveness and safety of Chinese patent medicine for mild-to-moderate active UC. In the included 33 RCTs, Chinese patent medicines were frequently combined with Mesalazine in the treatment of UC. The findings from network meta-analyses indicate that using Xilei powder plus Mesalazine generates statistically less incidence of adverse events than that of other interventions. Combined treatments including Danshen freeze-dried powder plus Mesalazine and Kangfuxin lotion plus Mesalazine were superior in reducing recurrence rate, and Kangfuxin lotion plus Mesalazine and Zhikang capsule plus Mesalazine were superior in the disappearance of tenesmus. Danshen injection plus Mesalazine leads to statistical improvements in Mayo score.

The overall quality of the included RCTs in this NMA is low. Most studies were judged as unclear risk of performance bias, detection bias, and reporting bias, because of inadequate information about allocation concealment, blinding, and protocol registration. In addition, the disease phase and severity were not clearly reported, which limited the number of studies to be included. These methodological defects of the included studies may affect the authenticity and reliability of our findings.

A previous NMA indicated that there is no statistical difference between the Chinese herbal injection combined with Mesalazine and Mesalazine alone groups in the treatment of mild-to-moderate UC for composite outcome total effective rate [14]. Our findings about the effects of Chinese patent medicines in combination with Mesalazine on safety and recurrence rate are consistent with the previous findings. In contrast, we found that Danshen injection plus Mesalazine was superior to Mesalazine in improving the Mayo score. The different findings might be due to the use of different outcomes. Instead of the “total effective rate,” which is a composite, dichotomous variable, with no specific definition/criteria for each level, we use the specific Mayo score as an outcome.

In this study, the network plot is star shaped and does not form a typical network structure, which is consistent with the findings of two previous studies [13, 14]. There is a lack of direct comparison among the interventions included in this study. As a result, it is impossible to detect the inconsistency between included studies. Our findings are consistent with that of two previous NMA. Meanwhile, the network plot does not form a typical closed loop, reducing the certainty of our analyses. The results need to be interpreted with caution.

### 4.2. Strength and Limitation

This is the first systematic review and NMA evaluating the relative safety and effectiveness of Chinese patent medicines for mild-to-moderate active UC. Given the lack of head-to-head studies comparing Chinese patent medicines in this population, the rank probability of this work could inform clinical practice.

However, there were several limitations to this study. First, the sample sizes of the included studies were insufficient to draw definitive conclusions with respect to certain Chinese patent medicines. Second, the inclusion and exclusion criteria of this study are limited to the clinical trials of Chinese patent medicine combined with Mesalazine compared with Mesalazine and placebo, and the selection of outcome indicators also limits the inclusion of more RCTs.

## 5. Conclusion

This is the first systematic review and NMA evaluating the relative effects and safety of Chinese patent medicine for mild-to-moderate active UC. Several Chinese patent medicines used alone or in combination with Mesalazine are more effective and safer than that of Mesalazine used alone in some outcomes, such as adverse events, recurrence rate, and disappearance rate of main clinical symptoms. However, none of the Chinese patent medicines or those combined with western medicines were found to have better efficacy than Mesalazine in all outcome indicators. Due to low methodological quality, no confirmative conclusion can be drawn. More future high-quality studies with direct comparisons of Chinese patent medicines are needed.

## Figures and Tables

**Table 1:** Search strategy in PubMed.

Search	Query
#1	Search: ((ulcerative colitis[Mesh Terms]) OR (ulcerative colitis[Title/Abstract])) OR (UC[Title/Abstract])
#2	Search: (((Chinese patent medicine[Mesh Terms]) OR (Chinese patent medicine[Title/Abstract])) OR (Chinese proprietary medicine [Mesh Terms])) OR (Chinese proprietary medicine [Title/Abstract])
#3	Search: ((((((((((((((((((((xileisan[Title/Abstract] OR (bawei xileisan[Title/Abstract])) OR (yunnan baiyao[Title/Abstract])) OR (fufangkushen colon-coated capsule[Title/Abstract])) OR (zhikang capsule[Title/Abstract])) OR (bupiyichang[Title/Abstract])) OR (yunnan hongyao[Title/Abstract])) OR (kangfuxinye[Title/Abstract])) OR (danshen injection[Title/Abstract])) OR (fengliaochangweikang[Title/Abstract])) OR (fufang huangbaiye[Title/Abstract])) OR (tongxiening[Title/Abstract])) OR (jiechangning[Title/Abstract])) OR (Hudi enteric-coated capsule[Title/Abstract])) OR (fuzilizhong[Title/Abstract])) OR (jinqiaomai[Title/Abstract])) OR (fufanggancao[Title/Abstract])) OR (shuangliaohoufeng[Title/Abstract])) OR (xianglian[Title/Abstract])) OR (gubenyichang[Title/Abstract])) OR (guchangzhixie[Title/Abstract]))
#4	Search: (((randomized controlled trial[Publication Type]) OR (clinical trial[Publication Type])) OR (randomized[Title/Abstract])) OR (randomly[Title/Abstract])
#5	Search: #2 OR #3
#6	Search: #1 AND #4 AND #5

**Table 2:** Characteristics of the included literatures (*n* = 33).

Study ID	Country	Intervention	Control	*N*(I/C)	Treatment duration (weeks)	Funding
Zhang [46]	China	Bupi Yichang pill, Mesalazine	Mesalazine	40/39	4	No
Yao [44]	China	ChangYanNing capsule, Mesalazine	Mesalazine	36/36	4	No
Liu [33]	China	ChangYanNing capsule, Mesalazine	Mesalazine	45/44	4	No
Luo [35]	China	ChangYanNing capsule, Mesalazine	Mesalazine	70/70	4	Yes
Wang [40]	China	Danshen freeze-dried powder, Mesalazine	Mesalazine	60/60	2.6	Yes
Yang [43]	China	Danshen injection, Mesalazine	Mesalazine	30/30	4	No
Deng [25]	China	Compound Huangbai liquid	Mesalazine	60/60	6	No
Liu [32]	China	Compound Sophora enteric capsules, placebo	Mesalazine, placebo	24/19	8	No
Shen [20]	China	Enteric-coated Hudi capsules, placebo, Mesalazine	Mesalazine	116/115 118/115	6	No
Wang [22]	China	JieChangNing, Mesalazine	Mesalazine	23/23	4	Yes
He [29]	China	Kangfuxin lotion, Mesalazine	Mesalazine	60/60	4	No
Zhang [45]	China	Kangfuxin lotion, Mesalazine, Yunnan Hongyao capsule	Kangfuxin lotion, Mesalazine	30/30	4	No
Pan [38]	China	Kangfuxin lotion, Mesalazine	Mesalazine	36/36	4	No
Bai [23]	China	Kangfuxin lotion, Mesalazine	Mesalazine	38/30	4	No
Liang [31]	China	Kangfuxin lotion, Mesalazine	Mesalazine	31/31	4	No
Li [30]	China	Kangfuxin lotion, Mesalazine	Mesalazine	36/36	4	No
Wen [41]	China	Kangfuxin lotion, Mesalazine	Mesalazine	55/55	4	Yes
Zheng [48]	China	Kangfuxin lotion, Mesalazine	Mesalazine	47/32	4	No
Gong [28]	China	Kangfuxin lotion, Mesalazine	Mesalazine	40/40	4	No
Gao [27]	China	Kangfuxin lotion, Mesalazine	Mesalazine	30/30	4	Yes
Ma [36]	China	Xilei powder, Yunnan white drug-powder, Shengji powder, Mesalazine	Mesalazine	26/20	12	No
Zhu [50]	China	Xilei powder, Mesalazine	Mesalazine	29/29	4	No
Zhang [47]	China	Yunnan white drug-powder, Mesalazine	Mesalazine	30/30	3	No
Deng [26]	China	Danshen injection, Mesalazine	Mesalazine	55/55	4	No
Ma [37]	China	Kangfuxin lotion, Mesalazine	Mesalazine	30/30	4	No
Yan [52]	China	Maintaining Intestines Antidiarrheal Pills, Mesalazine	Mesalazine	40/40	4	No
Liang [51]	China	Danshen injection, Mesalazine	Mesalazine	60/60	4	No
Zhu [49]	China	Xilei powder	Mesalazine	27/26	4	No
He [21]	China	Xilei powder	Mesalazine	15/15	2	Yes
Wang [39]	China	Zhikang capsule	Yunnan white drug-powder	30/30	3	No
Chen [24]	China	Zhikang capsule, Mesalazine	Mesalazine	32/26	2	No
Lu [34]	China	Aconitum Lizhong pill, Mesalazine	Mesalazine	60/60	8	No
Xu [42]	China	Shengmai injection, Mesalazine	Mesalazine	50/50	2	No

**Table 3:** Methodologic quality of the included studies.

Study	Random sequence generation	Allocation concealment	Blinding of participants and personnel	Blinding of outcome assessment	Incomplete outcome data	Selective reporting	Other bias
Zhang [46]	Unclear	Unclear	Unclear	Unclear	Low	Unclear	Unclear
Yao [44]	Low	Unclear	Unclear	Unclear	Low	Unclear	Unclear
Liu [33]	Unclear	High	Unclear	Unclear	Low	Unclear	Unclear
Luo [35]	Unclear	Unclear	Unclear	Unclear	Low	Unclear	Low
Wang [40]	Unclear	Unclear	Low	Unclear	Low	Unclear	Low
Yang [43]	Low	Unclear	Low	Low	Low	Unclear	Unclear
Deng [25]	Low	Unclear	Unclear	Unclear	Low	Unclear	Unclear
Liu [32]	Unclear	High	Low	Low	Low	Unclear	Unclear
Shen [20]	Low	Low	Low	Low	Low	Low	Unclear
Wang [22]	Low	Unclear	Low	Low	Low	Unclear	Low
He [29]	Low	Unclear	Unclear	Unclear	Low	Unclear	Unclear
Zhang [45]	Unclear	Unclear	Low	Low	Low	Unclear	Unclear
Pan [38]	Unclear	High	Low	Low	Low	Unclear	Unclear
Bai [23]	Unclear	Unclear	Unclear	Unclear	Low	Unclear	Unclear
Liang [31]	Low	Unclear	Unclear	Unclear	Low	Unclear	Unclear
Li [30]	Unclear	Unclear	Unclear	Unclear	Low	Unclear	Unclear
Wen [41]	Low	Unclear	Unclear	Unclear	Low	Unclear	Low
Zheng [48]	Unclear	Unclear	Unclear	Unclear	Low	Unclear	Unclear
Gong [28]	Low	Unclear	Unclear	Unclear	Low	Unclear	Unclear
Gao [27]	Low	Unclear	Unclear	Unclear	Low	Unclear	Low
Ma [36]	Low	Unclear	Unclear	Unclear	Low	Unclear	Unclear
Zhu [50]	Low	Unclear	Unclear	Unclear	Low	Unclear	Unclear
Zhang [47]	Unclear	Unclear	Unclear	Unclear	Low	Unclear	Unclear
Deng [26]	Low	Unclear	Unclear	Unclear	Low	Unclear	Unclear
Ma [37]	Unclear	Unclear	Unclear	Unclear	Low	Unclear	Unclear
Yan [52]	Unclear	Unclear	Unclear	Unclear	Low	Unclear	Unclear
Liang [51]	Unclear	Unclear	Unclear	Unclear	Low	Unclear	Unclear
Zhu [49]	Low	Unclear	Unclear	Unclear	Low	Unclear	Unclear
He [21]	Unclear	Unclear	Low	Unclear	Low	Unclear	Low
Wang [39]	Unclear	Unclear	Low	Low	Low	Unclear	Unclear
Chen [24]	Low	Unclear	Unclear	Unclear	Low	Unclear	Unclear
Lu [34]	Unclear	Unclear	Unclear	Unclear	Low	Unclear	Unclear
Xu [42]	Unclear	Unclear	Unclear	Unclear	Low	Unclear	Unclear

**Table 4:** Results of the pairwise meta-analysis.

Comparison	Adverse events		Recurrence rate		Disappearance of mucopurulent bloody stool		Disappearance of abdominal pain		Disappearance of diarrhea		Disappearance of tenesmus		Mayo score	
	*n*	RR [95% CI]	*n*	RR [95% CI]	*n*	RR [95% CI]	*n*	RR [95% CI]	*n*	RR [95% CI]	*n*	RR [95% CI]	*n*	MD [95% CI]
XLSplusM vs. M	1	1.45 [0.26, 7.99]	1	0.32 [0.07, 1.46]	1	1.34 [1.00, 1.80]	1	**1.32 [1.01, 1.72]**	1	1.36 [0.98, 1.89]	—		—	
XLS vs. M	2	0.14 [0.02, 1.05]	—		1	1.03 [0.74, 1.43]	1	1.21 [0.88, 1.66]	1	1.03 [0.72, 1.49]	—		—	
HDplusPLA vs. MplusPLA	—		—		1	**1.27 [1.02, 1.59]**	—		—		1	1.31 [0.98, 1.75]	—	
HDplusM vs. MplusPLA	—		—		1	**1.31 [1.06, 1.63]**	—		—		1	**1.38 [1.05, 1.80]**	—	
KFXplusM vs. M	6	1.19 [0.69, 2.04]	6	**0.29 [0.17, 0.51]**	1	**1.23 [1.01, 1.49]**	1	**1.25 [1.02, 1.54]**	1	**1.27 [1.01, 1.59]**	1	**1.31 [1.03, 1.68]**	—	
YNBY vs. ZK	—		1	0.53 [0.04, 7.50]	1	1.89 [1.01, 3.55]	1	1.70 [0.94, 3.08]	1	1.70 [0.94, 3.08]	1	1.89 [1.01, 3.55]	—	
BPYCplusM vs. M	1	0.33 [0.07, 1.51]	—		—		1	**2.47 [1.65, 3.70]**	1	**1.89 [1.32, 2.71]**	—		—	
ZKplusM vs. M	1	1.08 [0.27, 4.41]	—		1	1.36 [0.90, 2.06]	1	1.61 [0.90, 2.88]	1	1.53 [0.96, 2.42]	1	0.64 [0.30, 1.36]	—	
YNBYplusM vs. M	1	0.50 [0.10, 2.53]	—		1	1.42 [0.83, 2.44]	—		—		—		—	
DSFplusM vs. M	1	1.60 [0.56, 4.61]	1	**0.22 [0.09, 0.53]**	—		—		—		—		—	
DSIplusM vs. M	2	1.09 [0.50, 2.37]	1	0.55 [0.22, 1.37]	—		—		—		—		1	**−1.60 [−2.26, −0.94]**
JCNplusM vs. M	—		—		—		—		—		—		1	−0.22 [−1.19, 0.75]
FFHBplusM vs. M	1	1.50 [0.57, 3.95]	—		—		—		—		—		—	
FFKSplusPLA vs. MplusPLA	1	2.38 [0.27, 21.05]	—		—		—		—		—		—	
FZLZplusM vs. M	1	1.33 [0.31, 5.70]	—		—		—		—		—		—	
SMIplusM vs. M	1	0.83 [0.27, 2.55]	—		—		—		—		—		—	
CYNplusM vs. M	3	0.99 [0.36, 2.77]	—		—		—		—		—		—	
GCZXplusM vs. M	—		1	0.78 [0.52, 1.17]	—		—		—		—		—	
KFXplusM vs. YNHYplusKFXplusM	—		1	0.20 [0.01, 4.00]	—		—		—		—		—	
YNBYplusSJSplusXLSplusM vs. M	—		1	0.46 [0.12, 1.71]	—		—		—		—		—	

Significant results are in bold.

**Table 5:** Adverse events, recurrence rate, disappearance of mucopurulent bloody stool/abdominal pain/diarrhea/tenesmus, and Mayo score based on network meta-analysis.

*(a) Adverse events*												
XLSplusM												
**0.00 (0.00, 0.02)**	FZLZplusM											
**0.00 (0.00, 0.02)**	0.84 (0.04, 20.86)	XLS										
**0.00 (0.00, 0.10)**	5.53 (0.33, 133.79)	6.67 (0.31, 213.75)	BPYCplusM									
**0.00 (0.00, 0.02)**	1.31 (0.07, 22.05)	1.48 (0.06, 33.09)	0.23 (0.01, 3.90)	ZKplusM								
**0.00 (0.00, 0.02)**	1.37 (0.13, 15.57)	1.59 (0.11, 25.97)	0.26 (0.02, 2.64)	1.08 (0.09, 12.93)	CYNplusM							
**0.00 (0.00, 0.02)**	1.42 (0.19, 11.04)	1.63 (0.16, 21.02)	0.26 (0.02, 1.84)	1.12 (0.15, 9.04)	1.03 (0.28, 4.01)	M						
**0.00 (0.00, 0.03)**	1.78 (0.13, 26.89)	2.00 (0.11, 43.38)	0.32 (0.02, 4.61)	1.36 (0.10, 21.44)	1.32 (0.14, 12.25)	1.24 (0.21, 7.51)	SMIplusM					
**0.00 (0.00, 0.02)**	1.11 (0.13, 9.98)	1.35 (0.11, 18.09)	0.21 (0.02, 1.73)	0.91 (0.10, 8.17)	0.84 (0.18, 3.88)	0.80 (0.35, 1.79)	0.65 (0.09, 4.39)	KFXplusM				
**0.00 (0.00, 0.01)**	0.89 (0.07, 11.73)	1.00 (0.06, 19.30)	0.16 (0.01, 2.02)	0.66 (0.05, 9.93)	0.63 (0.08, 5.21)	0.62 (0.12, 3.13)	0.48 (0.04, 5.38)	0.77 (0.12, 4.85)	FFHBplusM			
**0.00 (0.00, 0.07)**	3.59 (0.17, 76.39)	3.98 (0.17, 125.54)	0.64 (0.02, 13.17)	2.69 (0.14, 65.55)	2.60 (0.20, 38.32)	2.48 (0.27, 26.88)	2.04 (0.12, 36.46)	3.05 (0.30, 38.01)	4.18 (0.26, 71.82)	YNBYplusM		
**0.00 (0.00, 0.02)**	1.26 (0.13, 14.65)	1.52 (0.11, 24.46)	0.24 (0.02, 2.44)	1.03 (0.10, 11.71)	0.95 (0.16, 5.77)	0.92 (0.27, 3.16)	0.73 (0.09, 6.22)	1.12 (0.27, 5.01)	1.49 (0.20, 11.78)	0.38 (0.03, 4.50)	DSIplusM	
**0.00 (0.00, 0.01)**	0.78 (0.06, 11.28)	0.90 (0.05, 17.74)	0.14 (0.01, 1.94)	0.61 (0.05, 9.39)	0.57 (0.07, 5.10)	0.56 (0.10, 3.07)	0.44 (0.04, 5.17)	0.68 (0.11, 4.74)	0.92 (0.09, 9.72)	0.22 (0.01, 3.68)	0.60 (0.07, 4.93)	DSFplusM

*(b) Recurrence rate*												
DSFplusM												
0.28 (0.02, 4.13)	DSIplusM											
0.26 (0.02, 3.25)	0.87 (0.07, 11.72)	GCZXplusM										
0.59 (0.08, 5.26)	1.96 (0.26, 19.28)	2.23 (0.33, 20.99)	KFXplusM									
**0.13 (0.02, 0.78)**	0.45 (0.07, 3.03)	0.51 (0.09, 3.32)	**0.24 (0.07, 0.57)**	M								
0.58 (0.03, 16.00)	2.07 (0.11, 62.18)	2.17 (0.12, 50.57)	1.05 (0.08, 14.13)	4.61 (0.44, 56.19)	XLSplusM							

*(c) Disappearance of mucopurulent bloody stool*												
YNBYplusM												
2.10 (0.32, 16.03)	M											
0.79 (0.05, 13.00)	0.38 (0.05, 2.82)	ZKplusM										
0.45 (0.02, 8.86)	0.22 (0.02, 1.85)	0.57 (0.03, 11.39)	XLSplusM									
1.91 (0.12, 34.79)	0.90 (0.12, 7.23)	2.43 (0.14, 44.25)	4.36 (0.22, 90.77)	XLS								

*(d) Disappearance of abdominal pain*												
M												
0.30 (0.03, 3.05)	ZKplusM											
0.16 (0.01, 1.86)	0.51 (0.01, 16.91)	XLSplusM										
0.40 (0.04, 4.63)	1.37 (0.05, 36.72)	2.68 (0.08, 97.04)	XLSplusM									

*(e) Disappearance of diarrhea*												
KFXplusM												
7.40 (0.52, 117.45)	M											
2.21 (0.07, 83.33)	0.31 (0.03, 3.26)	ZKplusM										
1.85 (0.05, 76.22)	0.26 (0.02, 2.94)	0.85 (0.02, 22.98)	XLSplusM									
6.31 (0.18, 268.20)	0.85 (0.08, 9.65)	2.75 (0.10, 79.92)	3.30 (0.11, 114.24)	XLS								

*(f) Disappearance of tenesmus*												
ZKplusM												
0.47 (0.01, 27.37)	M											
**0.00 (0.00, 0.02)**	**0.00 (0.00, 0.02)**	KFXplusM										

*(g) Mayo score*												
DSIplusM												
−1.36 (−4.42, 1.75)	JCNplusM											
**−1.59 (−3.73, 0.54)**	−0.22 (−2.46, 2.00)	M										

Significant results are in bold.

**Table 6:** Rank probabilities of each treatment in terms of adverse events (a), recurrence rate (b), disappearance of mucopurulent bloody stool (c), abdominal pain (d), diarrhea (e), tenesmus (f), and Mayo score (g) effect based on network meta-analysis.

*(a) Adverse events*													
Treatment	Rank 1	Rank 2	Rank 3	Rank 4	Rank 5	Rank 6	Rank 7	Rank 8	Rank 9	Rank 10	Rank 11	Rank 12	Rank 13

DSFplusM	0.2	0.17	0.14	0.1	0.09	0.06	0.05	0.05	0.05	0.04	0.03	0.01	0
DSIplusM	0.03	0.06	0.11	0.11	0.11	0.1	0.11	0.1	0.11	0.09	0.05	0.01	0
YNBYplusM	0.02	0.03	0.03	0.03	0.03	0.03	0.04	0.05	0.08	0.12	0.24	0.29	0
FFHBplusM	0.15	0.16	0.14	0.11	0.09	0.09	0.06	0.06	0.05	0.04	0.03	0.01	0
KFXplusM	0.02	0.06	0.11	0.15	0.15	0.14	0.13	0.1	0.08	0.04	0.01	0	0
SMIplusM	0.04	0.06	0.06	0.07	0.07	0.07	0.07	0.09	0.14	0.15	0.13	0.07	0
M	0	0	0.01	0.05	0.13	0.21	0.24	0.22	0.1	0.03	0	0	0
CYNplusM	0.04	0.06	0.09	0.12	0.09	0.09	0.1	0.11	0.12	0.11	0.06	0.02	0
ZKplusM	0.11	0.1	0.09	0.08	0.07	0.07	0.06	0.07	0.09	0.1	0.1	0.05	0
BPYCplusM	0	0.01	0.01	0.01	0.02	0.02	0.02	0.03	0.05	0.13	0.22	0.47	0
XLSplusM	0.23	0.15	0.09	0.07	0.07	0.05	0.05	0.05	0.07	0.07	0.06	0.04	0
FZLZplusM	0.16	0.13	0.1	0.08	0.08	0.06	0.07	0.06	0.07	0.08	0.06	0.03	0
XLS	0.00	0.00	0.00	0.00	0.00	0.00	0.00	0.00	0.00	0.00	0.00	0.00	0.99

*(b) Recurrence rate*													
Treatment	Rank 1			Rank 2		Rank 3		Rank 4		Rank 5		Rank 6	
DSFplusM	0.01			0.02		0.06		0.13		0.25		0.53	
DSIplusM	0.13			0.24		0.29		0.19		0.1		0.05	
GCZXplusM	0.15			0.31		0.3		0.14		0.07		0.03	
KFXplusM	0			0.03		0.15		0.36		0.34		0.12	
M	0.64			0.29		0.06		0.01		0		0	
XLSplusM	0.07			0.11		0.14		0.17		0.23		0.28	

*(c) Disappearance of mucopurulent bloody stool*													
Treatment				Rank 1		Rank 2		Rank 3		Rank 4		Rank 5	
YNBYplusM				0.17		0.28		0.28		0.15		0.12	
M				0		0.03		0.15		0.47		0.35	
ZKplusM				0.24		0.33		0.24		0.11		0.09	
XLSplusM				0.53		0.24		0.13		0.06		0.04	
XLS				0.06		0.13		0.19		0.22		0.4	

*(d) Disappearance of abdominal pain*													
Treatment						Rank 1		Rank 2		Rank 3		Rank 4	
M						0		0.05		0.29		0.66	
ZKplusM						0.26		0.38		0.25		0.11	
XLSplusM						0.55		0.27		0.13		0.05	
XLS						0.19		0.3		0.34		0.18	

*(e) Disappearance of diarrhea*													
Treatment				Rank 1		Rank 2		Rank 3		Rank 4		Rank 5	
KFXplusM				0.53		0.25		0.14		0.05		0.04	
M				0		0.01		0.12		0.45		0.41	
ZKplusM				0.18		0.3		0.31		0.13		0.09	
XLSplusM				0.24		0.33		0.25		0.1		0.08	
XLS				0.04		0.11		0.18		0.27		0.39	

*(f) Disappearance of tenesmus*													
Treatment								Rank 1		Rank 2		Rank 3	
KFXplusM								1		0		0	
M								0		0.7		0.3	
ZKplusM								0		0.3		0.7	

*(g) Mayo score*													
Treatment								Rank 1		Rank 2		Rank 3	
DSIplusM								0.04		0.12		0.84	
JCNplusM								0.39		0.48		0.13	
M								0.57		0.4		0.02	

## Data Availability

The data used to support the findings of this study are included within the article.

## Abbreviations

BPYC: Bupi Yichang pill
CYN: ChangYanNing capsule
DSF: Danshen freeze-dried powder
DFI: Danshen injection
FFHB: Compound Huangbai liquid
FZLZ: Aconitum Lizhong pill
GCZX: Maintaining Intestines Antidiarrheal Pills
HD: Enteric-coated Hudi capsules
JCN: JieChangNing
KFX: Kangfuxin lotion
KS: Compound Sophora enteric capsules
SJS: Shengji powder
SMI: Shengmai injection
XLS: Xilei powder
YNBY: Yunnan white drug-powder
YNHY: Yunnan Hong drug-powder
ZK: Zhikang capsule.

## Supplementary Materials

Supplementary Materials Supplementary S1: forest plot of pairwise meta-analysis of effect of Chinese patent medicine for mild-to-moderate active ulcerative colitis.

## References

[1] Danese S., Fiocchi C. (2011). Ulcerative colitis. *New England Journal of Medicine*.

[2] de Lange K. M., Moutsianas L., Lee J. C. (2017). Genome-wide association study implicates immune activation of multiple integrin genes in inflammatory bowel disease. *Nature Genetics*.

[3] Piovani D., Danese S., Peyrin-Biroulet L., Nikolopoulos G. K., Lytras T., Bonovas S. (2019). Environmental risk factors for inflammatory bowel diseases: an umbrella review of meta-analyses. *Gastroenterology*.

[4] Franzosa E. A., Sirota-Madi A., Avila-Pacheco J. (2019). Gut microbiome structure and metabolic activity in inflammatory bowel disease. *Nature Microbiology*.

[5] Neurath M. F. (2014). Cytokines in inflammatory bowel disease. *Nature Reviews Immunology*.

[6] Wen S. (1956). Analysis and discussion of 23 cases of ulcerative colitis. *People’s Military Surgeon*.

[7] Jiang X.-L., Cui H.-F. (2002). An analysis of 10218 ulcerative colitis cases in China. *World Journal of Gastroenterology*.

[8] Su Y., Zhao D. (2014). A case of granulocytopenia and secondary typhoid infection caused by sulfasalazine. *Journal of Hebei Medical University*.

[9] Li X., Liu L., Li Y. (2014). Drug-induced hypersensitivity syndrome induced by sulfasalazine: a case report. *Journal of Clinical Dermatology*.

[10] Huang X., Zhao W., Wang Y. (2012). A case of sulfasalazine-induced servere drug eruption. *Chinese Journal of Dermatovenereology*.

[11] Zhang D., Li F. (2007). Acute pancreatitis caused by sulfasalazine. *Adverse Drug Reactions Journal*.

[12] Wang X. (2008). Advantages and countermeasures of traditional Chinese and western medicine in treating ulcerative colitis. *Chinese Journal of Integrated Traditional and Western Medicine*.

[13] Feng J., Li J., Yang Y. (2017). Network meta-analysis on the comparison of the efficacy of five traditional Chinese medicine injections combined with sulfasalazine in the treatment of mild to moderate ulcerative colitis in active phase. *Journal of Guangdong Medical University*.

[14] Feng J., Li J., Yang Y. (2018). Network meta-analysis of the efficacy of traditional Chinese medicine injection combined with mesalazine in the treatment of active ulcerative colitis. *Journal of Wenzhou Medical University*.

[15] (2021). Cochrane handbook for systematic reviews of interventions. https://training.cochrane.org/handbook/current.

[16] Hutton B., Salanti G., Caldwell D. M. (2015). The PRISMA extension statement for reporting of systematic reviews incorporating network meta-analyses of health care interventions: checklist and explanations. *Annals of Internal Medicine*.

[17] Bressler B., Marshall J. K., Bernstein C. N. (2015). Clinical practice guidelines for the medical management of nonhospitalized ulcerative colitis: the Toronto consensus. *Gastroenterology*.

[18] Hu M.-L., Zheng G., Lin H., Yang M., Zhang Y.-D., Han J.-M. (2019). Network meta-analysis on the effect of desensitizing toothpastes on dentine hypersensitivity. *Journal of Dentistry*.

[19] Van Valkenhoef G., Lu G., de Brock B., Hillege H., Ades A. E., Welton N. J. (2012). Automating network meta-analysis. *Research Synthesis Methods*.

[20] Shen H., Zhu L., Hu N. (2019). Hudi enteric-coated capsule combined with mesalazine enteric-coated tablet for active ulcerative colitis: a multi-centre, randomized, double-blinded and double-simulated clinical study. *Chinese Journal of Integrated Traditional and Western Medicine*.

[21] He Y., Li C., Zhao W. (2011). Observation on the efficacy of Xilei powder in the treatment of ulcerative colitis. *Chinese Journal of Clinical Medicine*.

[22] Wang Z., Chen Y., Huang J. (2016). Effect of Jiechangning on intestinal microecology in patients with ulcerative colitis. *Guangdong Medical Journal*.

[23] Bai B., Huang G., Zhu L. (2012). Clinical effect of Kangfuxin liquid combined with mesalazine on 68 cases of ulcerative colitis. *West China Journal of Pharmaceutical Sciences*.

[24] Chen G., Yao H., Li S. (2007). Clinical observation on 32 cases of ulcerative colitis treated by Zhikang capsule combined with mezalazine. *Lishizhen Medicine and Materia Medica Research*.

[25] Deng T., Quan D., Wu B. (2016). Effect of compound Huangbai solution combined with oral mesalazine on ulcerative colitis, intestinal microflora and serum inflammatory factors. *China Journal of Modern Medicine*.

[26] Deng W.-J., Ma Y., Ma L. (2016). Mesalazine in combination with Danshen injection for treatment of ulcerative colitis: curative efficacy and effect on inflammatory factors and coagulation parameters. *World Chinese Journal of Digestology*.

[27] Gao X., Zhao T., Lu X. (2019). Efficacy and safety of mesalazine combined with kangfuxin liquid retention enema in the treatment of ulcerative colitis. *Medical Journal of the Chinese People Armed Police Forces*.

[28] Gong J., Yan C., Zhang L. (2015). Curative efficacy of mesalazine in combination with kangfuxin solution enema in treatment of ulcerative colitis and its effects on coagulation parameters. *Medical Journal of the Chinese People’s Armed Police Forces*.

[29] He J., Yu J., Shen H. (2020). Clinical effect of retention enema with kangfuxin liquid combined with mesalazine for ulcerative colitis and its effect on levels of TNF-*α*, TGF-*β* and MMP-1. *Journal of New Chinese Medicine*.

[30] Li Y., Liu Y., Xu Y. (2017). Effect of kangfuxin liquid combined with mesalazine on serum inflammatory factors and T lymphocyte subsets in patients with ulcerative colitis. *West China Journal of Pharmaceutical Sciences*.

[31] Liang W., Liang L. (2018). The efficacy of kangfuxin liquid combined with mesalazine enteric-coated tablets in the treatment of ulcerative colitis. *Strait Pharmaceutical Journal*.

[32] Liu H., Chen J., Xu P. (2012). Compound Kushen enteric-coated capsules in treating 24 clinical cases of ulcerative colitis. *Chinese Journal of Integrated Traditional and Western Medicine*.

[33] Liu H. (2014). The efficacy of Changyanning capsule combined with mesalazine in the treatment of ulcerative colitis. *Acta Chinese Medicine and Pharmacology*.

[34] Lu B., Shi R. (2019). Effect of mesalazine combined with Fuzilizhong pills on ulcerative colitis of spleen and kidney Yang deficiency and its effect on serum HIF-1*α* and SOCS-3 levels. *China Modern Doctor*.

[35] Luo R. (2019). Clinical efficacy of Changyanning capsule combined with mesalazine in treatment of ulcerative colitis. *Inner Mongolia Medical Journal*.

[36] Ma G., Li Y., Du J. (2016). Effect of oral administration of Mesalazine combined with retention enema in the treatment of ulcerative colitis. *Chinese Modern Medicine*.

[37] Ma J. (2014). Mesalazine combinated with kangfuxin for ulcerative colitis. *Chinese Journal of Geriatric Care*.

[38] Pan H., Zhu X., Ji F. (2017). Observation of kangfuxin liquid combined with mesalazine enema in the treatment of ulcerative colitis. *Heilongjiang Medicine and Pharmacy*.

[39] Wang T. (2012). *Capsule Retention Enema in Treatment of Ulcerative Colitis Clinical Curative Effect Observation*.

[40] Wang W., Wei S., Xing W. (2018). Effect of Danshen powder injection on D-dimer, PT, FIB and PLT in patients with ulcerative colitis. *Journal of Clinical and Experimental Medicine*.

[41] Wen Y., Shi L., Li J. (2018). Efficacy of kangfuxin liquid combined with mesalazine in the treatment of 55 cases of ulcerative colitis. *Chinese Journal of Ethnomedicine and Ethnopharmacy*.

[42] Xu X., Zhang K., Wang Y. (2008). Shengmai injection in adjuvant treatment of ulcerative colitis 50 cases. *Chinese Journal of Integrated Traditional and Western Medicine*.

[43] Yang L., Wang J. (2017). Effect observation of salvia miltiorrhiza combined with mezalazine on ulcerative colitis. *Clinical Medicine*.

[44] Yao Z. (2018). Oral administration of Changyanning capsule plus mesalazine in the treatment of ulcerative colitis: clinical study. *Chinese Journal of Coloproctology*.

[45] Zhang C., Zhang X. (2018). Clinical effect of kangfuxin solution mixed with Yunnan Hongyao capsules in the treatment of mild to moderate ulcerative colitis. *China Modern Medicine*.

[46] Zhang C., Mi Z., Tian W. (2012). 40 cases of ulcerative colitis treated by integrated traditional Chinese and western medicine. *China Pharmaceuticals*.

[47] Zhang L. (2010). *Clinical Observation of Ulcerative Colitis with Mezalazine Plus Yunnanbaiyao*.

[48] Zheng L., Ma J., Xing J. (2015). Clinical observation of kangfuxin liquid combined with mesalazine in the treatment of ulcerative colitis. *Chinese Journal of Integrated Traditional and Western Medicine*.

[49] Zhu Y., Xie H.-Z. (2008). Efficacy comparison of Xilei San and mesalazine enemas for active distal ulcerative colitis. *World Chinese Journal of Digestology*.

[50] Zhu Y., Xie H. (2009). Effect of Xi Lei powder enemas and mezalazines for active ulcerative colitis. *Journal of Xinjiang Medical University*.

[51] Liang T., Tang H., Chen H. (2017). Study on clinical effect of mezalazine combined with Danshen injection for treatment of ulcerative colitis and influences of on inflammatory factors and coagulation parameters. *Chinese Journal of Integrated Traditional and Western Medicine*.

[52] Yan W., Song L. (2017). Mesalazine suppository complicated with Guchang Zhixie wan in the treatment of ulcerative colitis: effect observation on 40 cases. *Chinese Journal of Coloproctology*.

