# Supplementary material for: Chinese Patent Medicine as Adjuvant for Mild-to-Moderate Active Ulcerative Colitis: A Network Meta-Analysis of Randomized Controlled Trials
Source: Evid Based Complement Alternat Med. 2021 Aug 26;2021:1075886. doi: 10.1155/2021/1075886 (PMC8413025; doi:10.1155/2021/1075886)
Supplement: Supplementary Materials — Supplementary S1: forest plot of pairwise meta-analysis of effect of Chinese patent medicine for mild-to-moderate active ulcerative colitis. [file 1075886.f1.pdf]

Supplementary S1.
Forest plot of pairwise meta-analysis of effect of Chinese patent medicine for mild-to-moderate active ulcerative colitis.

1 CYN+M vs M

1.1 Adverse events

Study or Subgroup	Experimental Events	Experimental Total	Control Events	Control Total	Weight	Risk Ratio M-H, Fixed, 95% CI	Risk of Bias A	B	C	D	E	F	G
LiuHJ 2014	3	45	2	44	28.8%	1.47 [0.26, 8.36]	+	-	?	?	+	?	?
LuoRJ 2019	2	70	4	70	57.0%	0.50 [0.09, 2.64]	?	?	?	?	+	?	+
YaoZY 2018	2	36	1	36	14.2%	2.00 [0.19, 21.09]	+	?	?	?	+	?	?
**Total (95% CI)**		**151**		**150**	**100.0%**	**0.99 [0.36, 2.77]**							
Total events	7		7										

Heterogeneity: Chi² = 1.18, df = 2 (P = 0.55); I² = 0%
Test for overall effect: Z = 0.02 (P = 0.99)

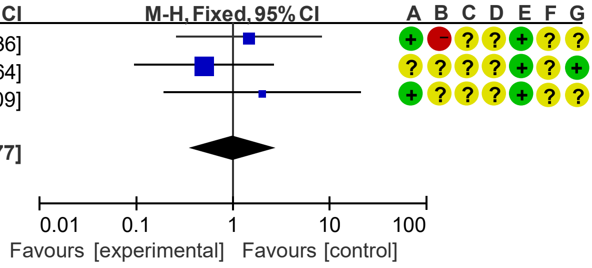

Risk of bias legend
(**A**) Random sequence generation (selection bias)
(**B**) Allocation concealment (selection bias)
(**C**) Blinding of participants and personnel (performance bias)
(**D**) Blinding of outcome assessment (detection bias)
(**E**) Incomplete outcome data (attrition bias)
(**F**) Selective reporting (reporting bias)
(**G**) Other bias

2 DSF+M vs M

2.1 Recurrence rate

Study or Subgroup	Experimental Events	Experimental Total	Control Events	Control Total	Weight	Risk Ratio M-H, Fixed, 95% CI	Risk of Bias A	B	C	D	E	F	G
WangWW 2018	5	60	23	60	100.0%	0.22 [0.09, 0.53]	?	?	+	?	+	?	+
**Total (95% CI)**		**60**		**60**	**100.0%**	**0.22 [0.09, 0.53]**							
Total events	5		23										

Heterogeneity: Not applicable
Test for overall effect: Z = 3.33 (P = 0.0009)

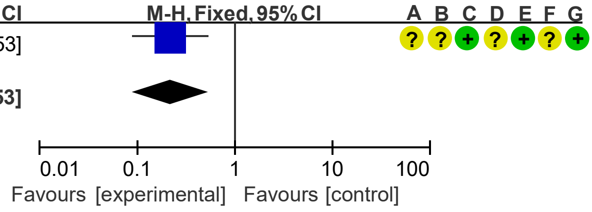

Risk of bias legend
(**A**) Random sequence generation (selection bias)
(**B**) Allocation concealment (selection bias)
(**C**) Blinding of participants and personnel (performance bias)
(**D**) Blinding of outcome assessment (detection bias)
(**E**) Incomplete outcome data (attrition bias)
(**F**) Selective reporting (reporting bias)
(**G**) Other bias

2.2 Adverse events

Study or Subgroup	Experimental Events	Experimental Total	Control Events	Control Total	Weight	Risk Ratio M-H, Fixed, 95% CI
WangWW 2018	8	60	5	60	100.0%	1.60 [0.56, 4.61]
**Total (95% CI)**		**60**		**60**	**100.0%**	**1.60 [0.56, 4.61]**
Total events	8		5			

Heterogeneity: Not applicable
Test for overall effect: Z = 0.87 (P = 0.38)

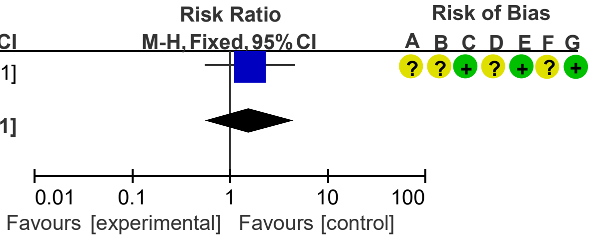

Risk of bias legend
(**A**) Random sequence generation (selection bias)
(**B**) Allocation concealment (selection bias)
(**C**) Blinding of participants and personnel (performance bias)
(**D**) Blinding of outcome assessment (detection bias)
(**E**) Incomplete outcome data (attrition bias)
(**F**) Selective reporting (reporting bias)
(**G**) Other bias

3 DSI+M vs M

3.1 Mayo

Study or Subgroup	Experimental Mean	Experimental SD	Experimental Total	Control Mean	Control SD	Control Total	Weight	Mean Difference IV, Fixed, 95% CI
YangLN 2017	1.8	1.4	30	3.4	1.2	30	100.0%	-1.60 [-2.26, -0.94]
**Total (95% CI)**			**30**			**30**	**100.0%**	**-1.60 [-2.26, -0.94]**

Heterogeneity: Not applicable
Test for overall effect: Z = 4.75 (P < 0.00001)

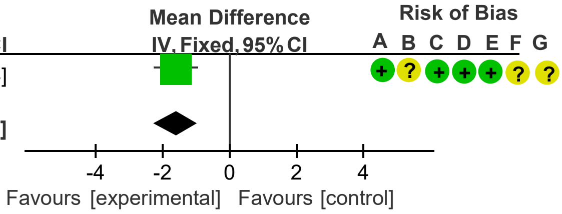

Risk of bias legend
(**A**) Random sequence generation (selection bias)
(**B**) Allocation concealment (selection bias)
(**C**) Blinding of participants and personnel (performance bias)
(**D**) Blinding of outcome assessment (detection bias)
(**E**) Incomplete outcome data (attrition bias)
(**F**) Selective reporting (reporting bias)
(**G**) Other bias

3.2 Adverse events

Study or Subgroup	Experimental Events	Experimental Total	Control Events	Control Total	Weight	Risk Ratio M-H, Fixed, 95% CI	Risk of Bias A B C D E F G
DengWJ 2016	4	55	6	55	54.5%	0.67 [0.20, 2.23]	+ ? ? ? + ? ?
LiangT 2017	8	60	5	60	45.5%	1.60 [0.56, 4.61]	? ? ? ? + ? ?
**Total (95% CI)**		**115**		**115**	**100.0%**	**1.09 [0.50, 2.37]**	
Total events	12		11				

Heterogeneity: Chi² = 1.14, df = 1 (P = 0.29); I² = 12%
Test for overall effect: Z = 0.22 (P = 0.83)

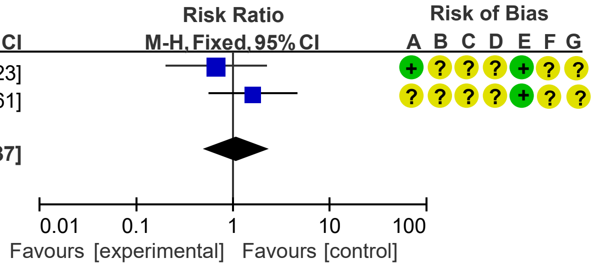

Risk of bias legend
(**A**) Random sequence generation (selection bias)
(**B**) Allocation concealment (selection bias)
(**C**) Blinding of participants and personnel (performance bias)
(**D**) Blinding of outcome assessment (detection bias)
(**E**) Incomplete outcome data (attrition bias)
(**F**) Selective reporting (reporting bias)
(**G**) Other bias

3.3 Recurrence rate

Study or Subgroup	Experimental Events	Experimental Total	Control Events	Control Total	Weight	Risk Ratio M-H, Fixed, 95% CI	Risk of Bias A B C D E F G
DengWJ 2016	6	55	11	55	100.0%	0.55 [0.22, 1.37]	+ ? ? ? + ? ?
**Total (95% CI)**		**55**		**55**	**100.0%**	**0.55 [0.22, 1.37]**	
Total events	6		11				

Heterogeneity: Not applicable
Test for overall effect: Z = 1.29 (P = 0.20)

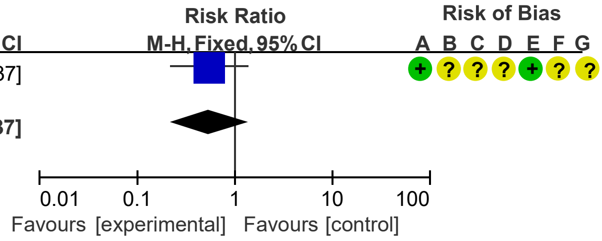

Risk of bias legend
(**A**) Random sequence generation (selection bias)
(**B**) Allocation concealment (selection bias)
(**C**) Blinding of participants and personnel (performance bias)
(**D**) Blinding of outcome assessment (detection bias)
(**E**) Incomplete outcome data (attrition bias)
(**F**) Selective reporting (reporting bias)
(**G**) Other bias

4 FFHB+M vs M

4.1 Adverse events

Study or Subgroup	Experimental Events	Experimental Total	Control Events	Control Total	Weight	Risk Ratio M-H, Fixed, 95% CI	Risk of Bias A	B	C	D	E	F	G
DengTY 2016	9	60	6	60	100.0%	1.50 [0.57, 3.95]	+	?	?	?	+	?	?
**Total (95% CI)**		**60**		**60**	**100.0%**	**1.50 [0.57, 3.95]**							
Total events	9		6										

Heterogeneity: Not applicable

Test for overall effect: Z = 0.82 (P = 0.41)

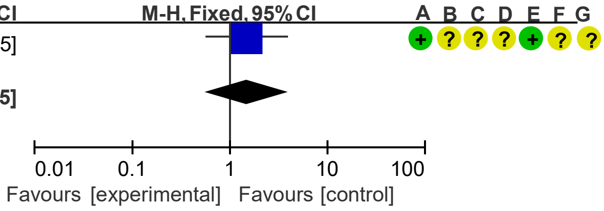

Risk of bias legend

(**A**) Random sequence generation (selection bias)
(**B**) Allocation concealment (selection bias)
(**C**) Blinding of participants and personnel (performance bias)
(**D**) Blinding of outcome assessment (detection bias)
(**E**) Incomplete outcome data (attrition bias)
(**F**) Selective reporting (reporting bias)
(**G**) Other bias

5 FFKS+placebo vs M+placebo

5.1 Adverse events

Study or Subgroup	Experimental Events	Experimental Total	Control Events	Control Total	Weight	Risk Ratio M-H, Fixed, 95% CI	Risk of Bias A	B	C	D	E	F	G
LiuHY 2012	3	24	1	19	100.0%	2.38 [0.27, 21.05]	?	-	+	+	+	?	?
**Total (95% CI)**		**24**		**19**	**100.0%**	**2.38 [0.27, 21.05]**							
Total events	3		1										

Heterogeneity: Not applicable

Test for overall effect: Z = 0.78 (P = 0.44)

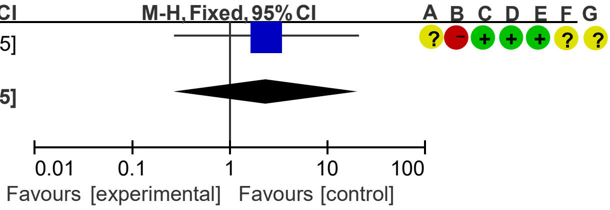

Risk of bias legend

(**A**) Random sequence generation (selection bias)
(**B**) Allocation concealment (selection bias)
(**C**) Blinding of participants and personnel (performance bias)
(**D**) Blinding of outcome assessment (detection bias)
(**E**) Incomplete outcome data (attrition bias)
(**F**) Selective reporting (reporting bias)
(**G**) Other bias

6 HD+placebo vs M+placebo
6.1 Disappearance of tenesmus

Study or Subgroup	Experimental Events	Experimental Total	Control Events	Control Total	Weight	Risk Ratio M-H, Fixed, 95% CI	Risk Ratio M-H, Fixed, 95% CI	Risk of Bias A B C D E F G
ShenH 2019	46	72	38	78	100.0%	1.31 [0.98, 1.75]		+ + + + + + ?
**Total (95% CI)**		**72**		**78**	**100.0%**	**1.31 [0.98, 1.75]**		
Total events	46		38					
Heterogeneity: Not applicable								
Test for overall effect: Z = 1.86 (P = 0.06)								

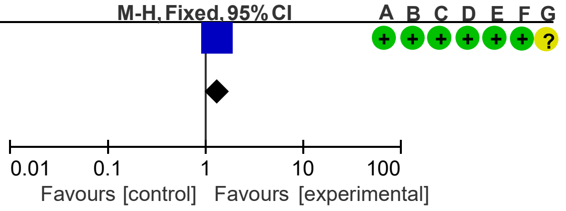

Risk of bias legend
(**A**) Random sequence generation (selection bias)
(**B**) Allocation concealment (selection bias)
(**C**) Blinding of participants and personnel (performance bias)
(**D**) Blinding of outcome assessment (detection bias)
(**E**) Incomplete outcome data (attrition bias)
(**F**) Selective reporting (reporting bias)
(**G**) Other bias

6.2 Disappearance of mucopurulent bloody stool

Study or Subgroup	Experimental Events	Experimental Total	Control Events	Control Total	Weight	Risk Ratio M-H, Fixed, 95% CI	Risk Ratio M-H, Fixed, 95% CI	Risk of Bias A B C D E F G
ShenH 2019	61	83	49	85	100.0%	1.27 [1.02, 1.59]		+ + + + + + ?
**Total (95% CI)**		**83**		**85**	**100.0%**	**1.27 [1.02, 1.59]**		
Total events	61		49					
Heterogeneity: Not applicable								
Test for overall effect: Z = 2.13 (P = 0.03)								

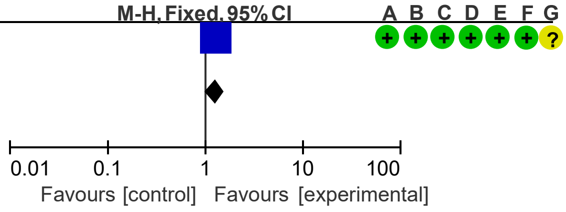

Risk of bias legend
(**A**) Random sequence generation (selection bias)
(**B**) Allocation concealment (selection bias)
(**C**) Blinding of participants and personnel (performance bias)
(**D**) Blinding of outcome assessment (detection bias)
(**E**) Incomplete outcome data (attrition bias)
(**F**) Selective reporting (reporting bias)
(**G**) Other bias

7 HD+M vs M+placebo

7.1 Disappearance of tenesmus

Study or Subgroup	Experimental Events	Experimental Total	Control Events	Control Total	Weight	Risk Ratio M-H, Fixed, 95% CI	Risk Ratio M-H, Fixed, 95% CI	Risk of Bias A B C D E F G
ShenH 2019	61	91	38	78	100.0%	1.38 [1.05, 1.80]		+ + + + + + ?
**Total (95% CI)**		**91**		**78**	**100.0%**	**1.38 [1.05, 1.80]**		
Total events	61		38					
Heterogeneity: Not applicable								
Test for overall effect: Z = 2.32 (P = 0.02)								

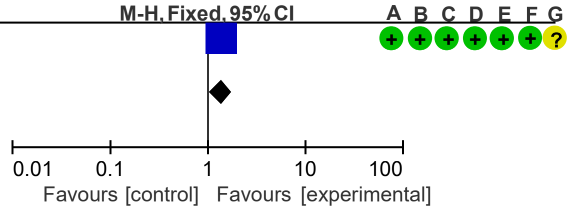

Risk of bias legend

(**A**) Random sequence generation (selection bias)
(**B**) Allocation concealment (selection bias)
(**C**) Blinding of participants and personnel (performance bias)
(**D**) Blinding of outcome assessment (detection bias)
(**E**) Incomplete outcome data (attrition bias)
(**F**) Selective reporting (reporting bias)
(**G**) Other bias

7.2 Disappearance of mucopurulent bloody stool

Study or Subgroup	Experimental Events	Experimental Total	Control Events	Control Total	Weight	Risk Ratio M-H, Fixed, 95% CI	Risk Ratio M-H, Fixed, 95% CI	Risk of Bias A B C D E F G
ShenH 2019	71	94	49	85	100.0%	1.31 [1.06, 1.63]		+ + + + + + ?
**Total (95% CI)**		**94**		**85**	**100.0%**	**1.31 [1.06, 1.63]**		
Total events	71		49					
Heterogeneity: Not applicable								
Test for overall effect: Z = 2.46 (P = 0.01)								

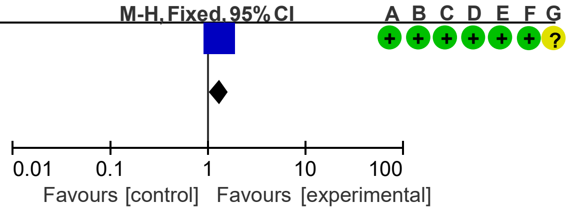

Risk of bias legend

(**A**) Random sequence generation (selection bias)
(**B**) Allocation concealment (selection bias)
(**C**) Blinding of participants and personnel (performance bias)
(**D**) Blinding of outcome assessment (detection bias)
(**E**) Incomplete outcome data (attrition bias)
(**F**) Selective reporting (reporting bias)
(**G**) Other bias

8 JCN+M vs M

8.1 Mayo

Study or Subgroup	Experimental Mean	Experimental SD	Experimental Total	Control Mean	Control SD	Control Total	Weight	Mean Difference IV, Fixed, 95% CI	Risk of Bias A	B	C	D	E	F	G
WangZJ 2016	3.3	1.84	23	3.52	1.49	23	100.0%	-0.22 [-1.19, 0.75]	+	?	+	+	+	?	+
**Total (95% CI)**			**23**			**23**	**100.0%**	**-0.22 [-1.19, 0.75]**							

Heterogeneity: Not applicable

Test for overall effect: Z = 0.45 (P = 0.66)

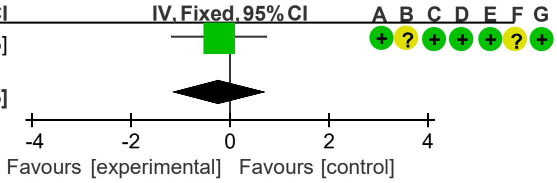

Risk of bias legend

(**A**) Random sequence generation (selection bias)

(**B**) Allocation concealment (selection bias)

(**C**) Blinding of participants and personnel (performance bias)

(**D**) Blinding of outcome assessment (detection bias)

(**E**) Incomplete outcome data (attrition bias)

(**F**) Selective reporting (reporting bias)

(**G**) Other bias

9 KFX+M vs M

9.1 Adverse events

Study or Subgroup	Experimental Events	Experimental Total	Control Events	Control Total	Weight	Risk Ratio M-H, Fixed, 95% CI	Risk of Bias A	B	C	D	E	F	G
GaoXK 2019	3	30	5	30	23.3%	0.60 [0.16, 2.29]	+	?	?	?	+	?	+
GongJQ 2015	5	40	3	40	14.0%	1.67 [0.43, 6.51]	+	?	?	?	+	?	?
HeJF 2020	10	60	8	60	37.2%	1.25 [0.53, 2.95]	+	?	?	?	+	?	?
LiangWD 2018	2	31	3	31	14.0%	0.67 [0.12, 3.72]	+	?	?	?	+	?	?
LiY 2017	4	36	2	36	9.3%	2.00 [0.39, 10.24]	?	?	?	?	+	?	?
MaJ 2014	1	30	0	30	2.3%	3.00 [0.13, 70.83]	?	?	?	?	+	?	?
**Total (95% CI)**		**227**		**227**	**100.0%**	**1.19 [0.69, 2.04]**							
Total events	25		21										

Heterogeneity: $Chi^2 = 2.40$, df = 5 (P = 0.79); $I^2 = 0\%$

Test for overall effect: Z = 0.62 (P = 0.54)

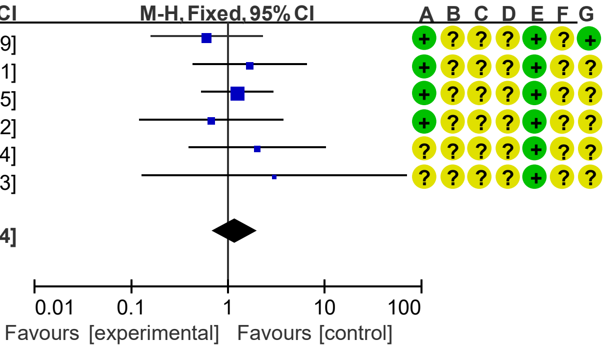

Risk of bias legend

(**A**) Random sequence generation (selection bias)

(**B**) Allocation concealment (selection bias)

(**C**) Blinding of participants and personnel (performance bias)

(**D**) Blinding of outcome assessment (detection bias)

(**E**) Incomplete outcome data (attrition bias)

(**F**) Selective reporting (reporting bias)

(**G**) Other bias

9.2 Recurrence rate

Study or Subgroup	Experimental Events	Experimental Total	Control Events	Control Total	Weight	Risk Ratio M-H, Fixed, 95% CI	A	B	C	D	E	F	G
BaiBJ 2012	3	38	7	30	16.2%	0.34 [0.10, 1.20]	?	?	?	?	+	?	?
GongJQ 2015	3	40	11	40	22.8%	0.27 [0.08, 0.90]	+	?	?	?	+	?	?
LiY 2017	2	36	5	36	10.3%	0.40 [0.08, 1.93]	?	?	?	?	+	?	?
MaJ 2014	2	30	6	30	12.4%	0.33 [0.07, 1.52]	?	?	?	?	+	?	?
PanHX 2017	0	36	3	36	7.2%	0.14 [0.01, 2.67]	?	-	+	+	+	?	?
WenY 2018	4	55	15	55	31.0%	0.27 [0.09, 0.75]	+	?	?	?	+	?	+
**Total (95% CI)**		**235**		**227**	**100.0%**	**0.29 [0.17, 0.51]**							
Total events	14		47										

Heterogeneity: Chi² = 0.50, df = 5 (P = 0.99); I² = 0%
Test for overall effect: Z = 4.32 (P < 0.0001)

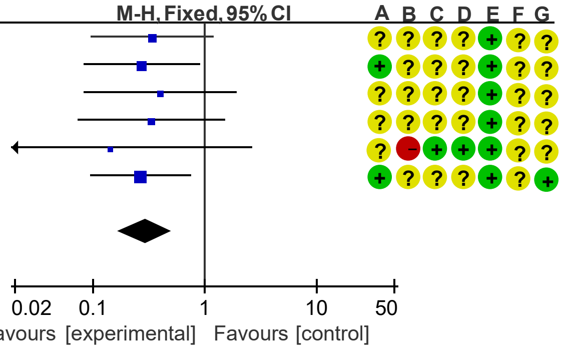

Risk of bias legend
(**A**) Random sequence generation (selection bias)
(**B**) Allocation concealment (selection bias)
(**C**) Blinding of participants and personnel (performance bias)
(**D**) Blinding of outcome assessment (detection bias)
(**E**) Incomplete outcome data (attrition bias)
(**F**) Selective reporting (reporting bias)
(**G**) Other bias

9.3 Disappearance of abdominal pain

Study or Subgroup	Experimental Events	Experimental Total	Control Events	Control Total	Weight	Risk Ratio M-H, Fixed, 95% CI	A	B	C	D	E	F	G
ZhengLJ 2015	40	41	21	27	100.0%	1.25 [1.02, 1.54]	?	?	?	?	+	?	?
**Total (95% CI)**		**41**		**27**	**100.0%**	**1.25 [1.02, 1.54]**							
Total events	40		21										

Heterogeneity: Not applicable
Test for overall effect: Z = 2.14 (P = 0.03)

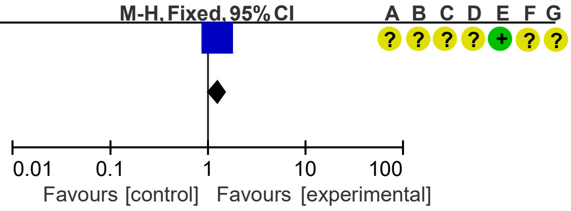

Risk of bias legend
(**A**) Random sequence generation (selection bias)
(**B**) Allocation concealment (selection bias)
(**C**) Blinding of participants and personnel (performance bias)
(**D**) Blinding of outcome assessment (detection bias)
(**E**) Incomplete outcome data (attrition bias)
(**F**) Selective reporting (reporting bias)
(**G**) Other bias

9.4 Disappearance of diarrhea

Study or Subgroup	Experimental Events	Experimental Total	Control Events	Control Total	Weight	Risk Ratio M-H, Fixed, 95% CI	Risk of Bias A	B	C	D	E	F	G
ZhengLJ 2015	38	40	21	28	100.0%	1.27 [1.01, 1.59]	?	?	?	?	+	?	?
**Total (95% CI)**		**40**		**28**	**100.0%**	**1.27 [1.01, 1.59]**							
Total events	38		21										
Heterogeneity: Not applicable													
Test for overall effect: Z = 2.06 (P = 0.04)													

Risk Ratio M-H, Fixed, 95% CI

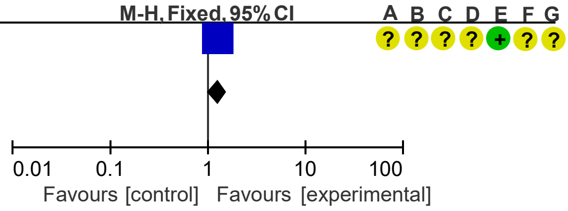

Risk of bias legend
(**A**) Random sequence generation (selection bias)
(**B**) Allocation concealment (selection bias)
(**C**) Blinding of participants and personnel (performance bias)
(**D**) Blinding of outcome assessment (detection bias)
(**E**) Incomplete outcome data (attrition bias)
(**F**) Selective reporting (reporting bias)
(**G**) Other bias

9.5 Disappearance of tenesmus

Study or Subgroup	Experimental Events	Experimental Total	Control Events	Control Total	Weight	Risk Ratio M-H, Fixed, 95% CI	Risk of Bias A	B	C	D	E	F	G
ZhengLJ 2015	34	34	16	21	100.0%	1.31 [1.03, 1.68]	?	?	?	?	+	?	?
**Total (95% CI)**		**34**		**21**	**100.0%**	**1.31 [1.03, 1.68]**							
Total events	34		16										
Heterogeneity: Not applicable													
Test for overall effect: Z = 2.19 (P = 0.03)													

Risk Ratio M-H, Fixed, 95% CI

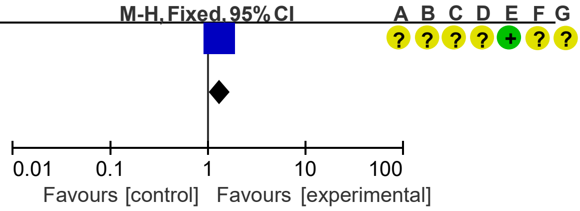

Risk of bias legend
(**A**) Random sequence generation (selection bias)
(**B**) Allocation concealment (selection bias)
(**C**) Blinding of participants and personnel (performance bias)
(**D**) Blinding of outcome assessment (detection bias)
(**E**) Incomplete outcome data (attrition bias)
(**F**) Selective reporting (reporting bias)
(**G**) Other bias

9.6 Disappearance of mucopurulent bloody stool

Study or Subgroup	Experimental Events	Experimental Total	Control Events	Control Total	Weight	Risk Ratio M-H, Fixed, 95% CI	A	B	C	D	E	F	G
ZhengLJ 2015	39	40	23	29	100.0%	1.23 [1.01, 1.49]	?	?	?	?	+	?	?
**Total (95% CI)**		**40**		**29**	**100.0%**	**1.23 [1.01, 1.49]**							
Total events	39		23										

Heterogeneity: Not applicable
Test for overall effect: Z = 2.10 (P = 0.04)

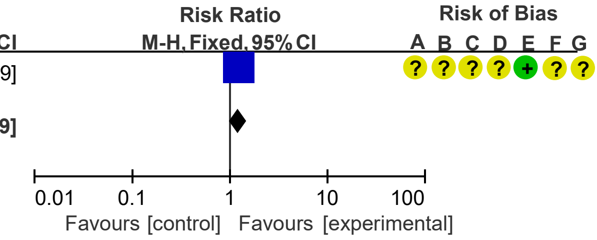

Risk of bias legend
(**A**) Random sequence generation (selection bias)
(**B**) Allocation concealment (selection bias)
(**C**) Blinding of participants and personnel (performance bias)
(**D**) Blinding of outcome assessment (detection bias)
(**E**) Incomplete outcome data (attrition bias)
(**F**) Selective reporting (reporting bias)
(**G**) Other bias

10 KFX+M vs YNHY+KFX+M

10.1 Recurrence rate

Study or Subgroup	Experimental Events	Experimental Total	Control Events	Control Total	Weight	Risk Ratio M-H, Fixed, 95% CI	A	B	C	D	E	F	G
ZhangC 2018	0	30	2	30	100.0%	0.20 [0.01, 4.00]	?	?	+	+	+	?	?
**Total (95% CI)**		**30**		**30**	**100.0%**	**0.20 [0.01, 4.00]**							
Total events	0		2										

Heterogeneity: Not applicable
Test for overall effect: Z = 1.05 (P = 0.29)

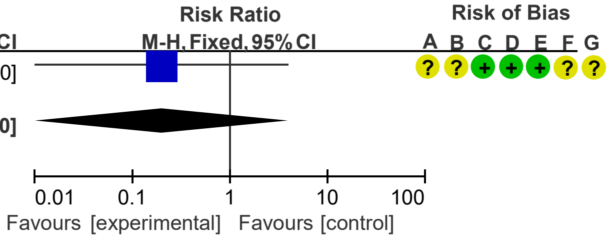

Risk of bias legend
(**A**) Random sequence generation (selection bias)
(**B**) Allocation concealment (selection bias)
(**C**) Blinding of participants and personnel (performance bias)
(**D**) Blinding of outcome assessment (detection bias)
(**E**) Incomplete outcome data (attrition bias)
(**F**) Selective reporting (reporting bias)
(**G**) Other bias

11 YNBY+SJS+XLS+M vs M

11.1 Recurrence rate

Study or Subgroup	Experimental Events	Experimental Total	Control Events	Control Total	Weight	Risk Ratio M-H, Fixed, 95% CI	Risk of Bias A	B	C	D	E	F	G
MaGP 2016	3	26	5	20	100.0%	0.46 [0.12, 1.71]	+	?	?	?	+	?	?
**Total (95% CI)**		**26**		**20**	**100.0%**	**0.46 [0.12, 1.71]**							
Total events	3		5										

Heterogeneity: Not applicable

Test for overall effect: Z = 1.16 (P = 0.25)

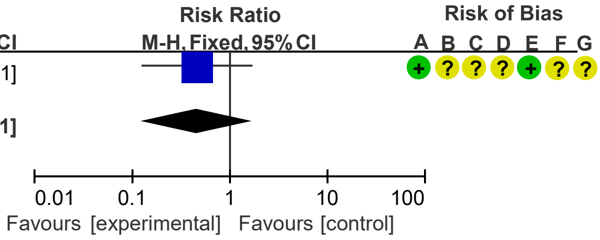

Risk of bias legend

(**A**) Random sequence generation (selection bias)
(**B**) Allocation concealment (selection bias)
(**C**) Blinding of participants and personnel (performance bias)
(**D**) Blinding of outcome assessment (detection bias)
(**E**) Incomplete outcome data (attrition bias)
(**F**) Selective reporting (reporting bias)
(**G**) Other bias

12 XLS+M vs M

12.1 Adverse events

Study or Subgroup	Experimental Events	Experimental Total	Control Events	Control Total	Weight	Risk Ratio M-H, Fixed, 95% CI	Risk of Bias A	B	C	D	E	F	G
ZhuY 2009	3	28	2	27	100.0%	1.45 [0.26, 7.99]	+	?	?	?	+	?	?
**Total (95% CI)**		**28**		**27**	**100.0%**	**1.45 [0.26, 7.99]**							
Total events	3		2										

Heterogeneity: Not applicable

Test for overall effect: Z = 0.42 (P = 0.67)

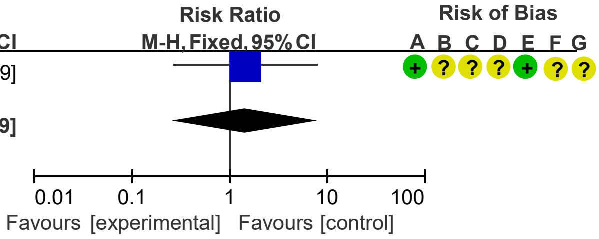

Risk of bias legend

(**A**) Random sequence generation (selection bias)
(**B**) Allocation concealment (selection bias)
(**C**) Blinding of participants and personnel (performance bias)
(**D**) Blinding of outcome assessment (detection bias)
(**E**) Incomplete outcome data (attrition bias)
(**F**) Selective reporting (reporting bias)
(**G**) Other bias

12.2 Disappearance of abdominal pain

Study or Subgroup	Experimental Events	Experimental Total	Control Events	Control Total	Weight	Risk Ratio M-H, Fixed, 95% CI	A	B	C	D	E	F	G
ZhuY 2009	26	28	19	27	100.0%	1.32 [1.01, 1.72]	+	?	?	?	+	?	?
**Total (95% CI)**		**28**		**27**	**100.0%**	**1.32 [1.01, 1.72]**							
Total events	26		19										

Heterogeneity: Not applicable
Test for overall effect: Z = 2.05 (P = 0.04)

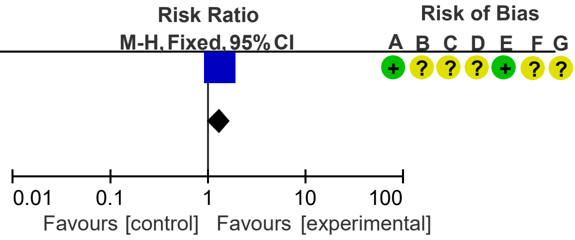

Risk of bias legend
(**A**) Random sequence generation (selection bias)
(**B**) Allocation concealment (selection bias)
(**C**) Blinding of participants and personnel (performance bias)
(**D**) Blinding of outcome assessment (detection bias)
(**E**) Incomplete outcome data (attrition bias)
(**F**) Selective reporting (reporting bias)
(**G**) Other bias

12.3 Disappearance of diarrhea

Study or Subgroup	Experimental Events	Experimental Total	Control Events	Control Total	Weight	Risk Ratio M-H, Fixed, 95% CI	A	B	C	D	E	F	G
ZhuY 2009	24	28	17	27	100.0%	1.36 [0.98, 1.89]	+	?	?	?	+	?	?
**Total (95% CI)**		**28**		**27**	**100.0%**	**1.36 [0.98, 1.89]**							
Total events	24		17										

Heterogeneity: Not applicable
Test for overall effect: Z = 1.85 (P = 0.06)

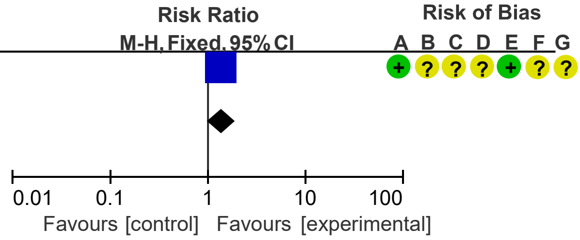

Risk of bias legend
(**A**) Random sequence generation (selection bias)
(**B**) Allocation concealment (selection bias)
(**C**) Blinding of participants and personnel (performance bias)
(**D**) Blinding of outcome assessment (detection bias)
(**E**) Incomplete outcome data (attrition bias)
(**F**) Selective reporting (reporting bias)
(**G**) Other bias

12.4 Recurrence rate

Study or Subgroup	Experimental Events	Experimental Total	Control Events	Control Total	Weight	Risk Ratio M-H, Fixed, 95% CI	Risk of Bias A	B	C	D	E	F	G
ZhuY 2009	2	28	6	27	100.0%	0.32 [0.07, 1.46]	+	?	?	?	+	?	?
**Total (95% CI)**		**28**		**27**	**100.0%**	**0.32 [0.07, 1.46]**							
Total events	2		6										

Heterogeneity: Not applicable
Test for overall effect: Z = 1.47 (P = 0.14)

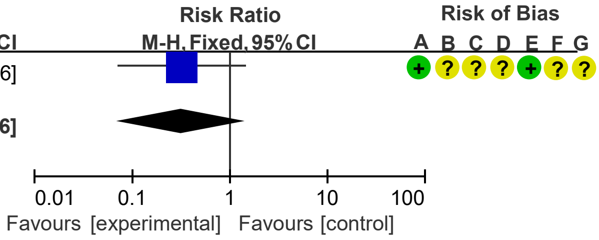

Risk of bias legend
(**A**) Random sequence generation (selection bias)
(**B**) Allocation concealment (selection bias)
(**C**) Blinding of participants and personnel (performance bias)
(**D**) Blinding of outcome assessment (detection bias)
(**E**) Incomplete outcome data (attrition bias)
(**F**) Selective reporting (reporting bias)
(**G**) Other bias

12.5 Disappearance of mucopurulent bloody stool

Study or Subgroup	Experimental Events	Experimental Total	Control Events	Control Total	Weight	Risk Ratio M-H, Fixed, 95% CI	Risk of Bias A	B	C	D	E	F	G
ZhuY 2009	25	28	18	27	100.0%	1.34 [1.00, 1.80]	+	?	?	?	+	?	?
**Total (95% CI)**		**28**		**27**	**100.0%**	**1.34 [1.00, 1.80]**							
Total events	25		18										

Heterogeneity: Not applicable
Test for overall effect: Z = 1.93 (P = 0.05)

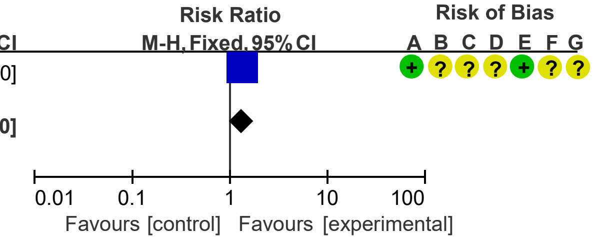

Risk of bias legend
(**A**) Random sequence generation (selection bias)
(**B**) Allocation concealment (selection bias)
(**C**) Blinding of participants and personnel (performance bias)
(**D**) Blinding of outcome assessment (detection bias)
(**E**) Incomplete outcome data (attrition bias)
(**F**) Selective reporting (reporting bias)
(**G**) Other bias

13 YNBY+M vs M

13.1 Adverse events

Study or Subgroup	Experimental Events	Experimental Total	Control Events	Control Total	Weight	Risk Ratio M-H, Fixed, 95% CI	Risk of Bias A	B	C	D	E	F	G
ZhangLL 2010	2	30	4	30	100.0%	0.50 [0.10, 2.53]	?	?	?	?	+	?	?
**Total (95% CI)**		**30**		**30**	**100.0%**	**0.50 [0.10, 2.53]**							
Total events	2		4										

Heterogeneity: Not applicable
Test for overall effect: Z = 0.84 (P = 0.40)

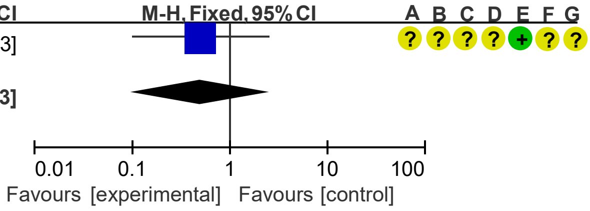

Risk of bias legend
(**A**) Random sequence generation (selection bias)
(**B**) Allocation concealment (selection bias)
(**C**) Blinding of participants and personnel (performance bias)
(**D**) Blinding of outcome assessment (detection bias)
(**E**) Incomplete outcome data (attrition bias)
(**F**) Selective reporting (reporting bias)
(**G**) Other bias

13.2 Disappearance of mucopurulent bloody stool

Study or Subgroup	Experimental Events	Experimental Total	Control Events	Control Total	Weight	Risk Ratio M-H, Fixed, 95% CI	Risk of Bias A	B	C	D	E	F	G
ZhangLL 2010	15	24	11	25	100.0%	1.42 [0.83, 2.44]	?	?	?	?	+	?	?
**Total (95% CI)**		**24**		**25**	**100.0%**	**1.42 [0.83, 2.44]**							
Total events	15		11										

Heterogeneity: Not applicable
Test for overall effect: Z = 1.27 (P = 0.20)

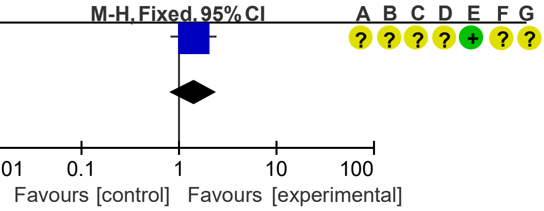

Risk of bias legend
(**A**) Random sequence generation (selection bias)
(**B**) Allocation concealment (selection bias)
(**C**) Blinding of participants and personnel (performance bias)
(**D**) Blinding of outcome assessment (detection bias)
(**E**) Incomplete outcome data (attrition bias)
(**F**) Selective reporting (reporting bias)
(**G**) Other bias

14 GCZX+M vs M

14.1 Recurrence rate

Study or Subgroup	Experimental Events	Experimental Total	Control Events	Control Total	Weight	Risk Ratio M-H, Fixed, 95% CI	A	B	C	D	E	F	G
YanWP 2017	18	35	23	35	100.0%	0.78 [0.52, 1.17]	?	?	?	?	+	?	?
**Total (95% CI)**		**35**		**35**	**100.0%**	**0.78 [0.52, 1.17]**							
Total events	18		23										

Heterogeneity: Not applicable

Test for overall effect: Z = 1.20 (P = 0.23)

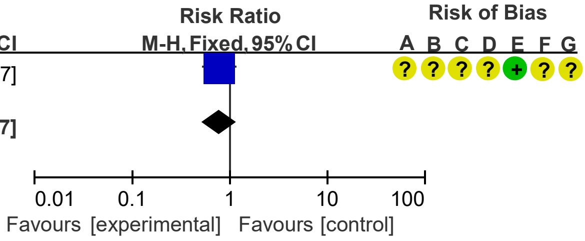

Risk of bias legend

(**A**) Random sequence generation (selection bias)

(**B**) Allocation concealment (selection bias)

(**C**) Blinding of participants and personnel (performance bias)

(**D**) Blinding of outcome assessment (detection bias)

(**E**) Incomplete outcome data (attrition bias)

(**F**) Selective reporting (reporting bias)

(**G**) Other bias

15 XLS vs M

15.1 Adverse events

Study or Subgroup	Experimental Events	Experimental Total	Control Events	Control Total	Weight	Risk Ratio M-H, Fixed, 95% CI	A	B	C	D	E	F	G
HeY 2011	0	15	1	15	20.8%	0.33 [0.01, 7.58]	?	?	+	?	+	?	+
ZhuY 2008	0	25	5	23	79.2%	0.08 [0.00, 1.44]	+	?	?	?	+	?	?
**Total (95% CI)**		**40**		**38**	**100.0%**	**0.14 [0.02, 1.05]**							
Total events	0		6										

Heterogeneity: Chi² = 0.43, df = 1 (P = 0.51); I² = 0%

Test for overall effect: Z = 1.92 (P = 0.06)

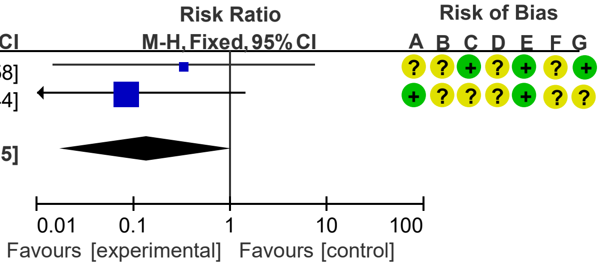

Risk of bias legend

(**A**) Random sequence generation (selection bias)

(**B**) Allocation concealment (selection bias)

(**C**) Blinding of participants and personnel (performance bias)

(**D**) Blinding of outcome assessment (detection bias)

(**E**) Incomplete outcome data (attrition bias)

(**F**) Selective reporting (reporting bias)

(**G**) Other bias

15.2 Disappearance of abdominal pain

Study or Subgroup	Experimental Events	Experimental Total	Control Events	Control Total	Weight	Risk Ratio M-H, Fixed, 95% CI	Risk of Bias A	B	C	D	E	F	G
ZhuY 2008	21	25	16	23	100.0%	1.21 [0.88, 1.66]	+	?	?	?	+	?	?
**Total (95% CI)**		**25**		**23**	**100.0%**	**1.21 [0.88, 1.66]**							
Total events	21		16										

Heterogeneity: Not applicable
Test for overall effect: Z = 1.16 (P = 0.25)

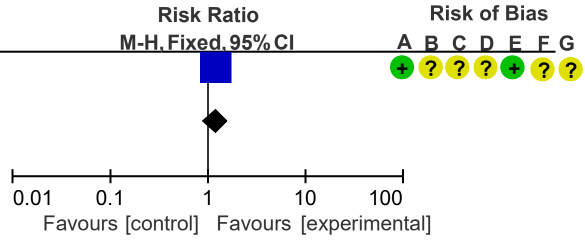

Risk of bias legend
(**A**) Random sequence generation (selection bias)
(**B**) Allocation concealment (selection bias)
(**C**) Blinding of participants and personnel (performance bias)
(**D**) Blinding of outcome assessment (detection bias)
(**E**) Incomplete outcome data (attrition bias)
(**F**) Selective reporting (reporting bias)
(**G**) Other bias

15.3 Disappearance of diarrhea

Study or Subgroup	Experimental Events	Experimental Total	Control Events	Control Total	Weight	Risk Ratio M-H, Fixed, 95% CI	Risk of Bias A	B	C	D	E	F	G
ZhuY 2008	18	25	16	23	100.0%	1.03 [0.72, 1.49]	+	?	?	?	+	?	?
**Total (95% CI)**		**25**		**23**	**100.0%**	**1.03 [0.72, 1.49]**							
Total events	18		16										

Heterogeneity: Not applicable
Test for overall effect: Z = 0.19 (P = 0.85)

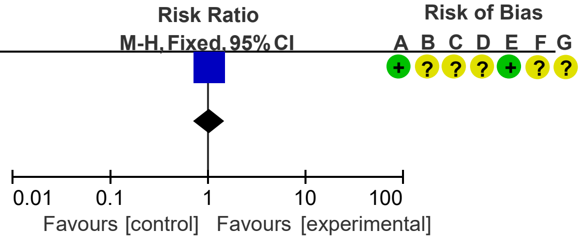

Risk of bias legend
(**A**) Random sequence generation (selection bias)
(**B**) Allocation concealment (selection bias)
(**C**) Blinding of participants and personnel (performance bias)
(**D**) Blinding of outcome assessment (detection bias)
(**E**) Incomplete outcome data (attrition bias)
(**F**) Selective reporting (reporting bias)
(**G**) Other bias

15.4 Disappearance of mucopurulent bloody stool

Study or Subgroup	Experimental Events	Experimental Total	Control Events	Control Total	Weight	Risk Ratio M-H, Fixed, 95% CI	Risk of Bias A B C D E F G
ZhuY 2008	19	25	17	23	100.0%	1.03 [0.74, 1.43]	+ ? ? ? + ? ?
**Total (95% CI)**		**25**		**23**	**100.0%**	**1.03 [0.74, 1.43]**	
Total events	19		17				

Heterogeneity: Not applicable
Test for overall effect: Z = 0.17 (P = 0.87)

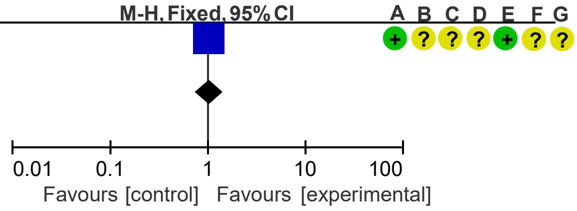

Risk of bias legend
(**A**) Random sequence generation (selection bias)
(**B**) Allocation concealment (selection bias)
(**C**) Blinding of participants and personnel (performance bias)
(**D**) Blinding of outcome assessment (detection bias)
(**E**) Incomplete outcome data (attrition bias)
(**F**) Selective reporting (reporting bias)
(**G**) Other bias

16 YNBY vs ZK

16.1 Disappearance of abdominal pain

Study or Subgroup	Experimental Events	Experimental Total	Control Events	Control Total	Weight	Risk Ratio M-H, Fixed, 95% CI	Risk of Bias A B C D E F G
WangT 2012	17	30	10	30	100.0%	1.70 [0.94, 3.08]	? ? + + + ? ?
**Total (95% CI)**		**30**		**30**	**100.0%**	**1.70 [0.94, 3.08]**	
Total events	17		10				

Heterogeneity: Not applicable
Test for overall effect: Z = 1.75 (P = 0.08)

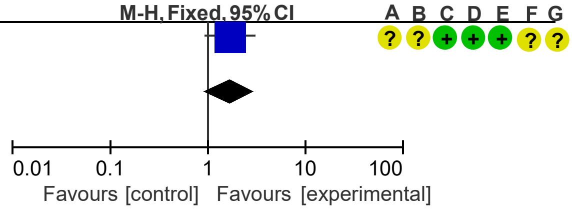

Risk of bias legend
(**A**) Random sequence generation (selection bias)
(**B**) Allocation concealment (selection bias)
(**C**) Blinding of participants and personnel (performance bias)
(**D**) Blinding of outcome assessment (detection bias)
(**E**) Incomplete outcome data (attrition bias)
(**F**) Selective reporting (reporting bias)
(**G**) Other bias

16.2 Disappearance of diarrhea

Study or Subgroup	Experimental Events	Experimental Total	Control Events	Control Total	Weight	Risk Ratio M-H, Fixed, 95% CI	Risk of Bias A	B	C	D	E	F	G
WangT 2012	17	30	10	30	100.0%	1.70 [0.94, 3.08]	?	?	+	+	+	?	?
**Total (95% CI)**		**30**		**30**	**100.0%**	**1.70 [0.94, 3.08]**							
Total events	17		10										
Heterogeneity: Not applicable													
Test for overall effect: Z = 1.75 (P = 0.08)													

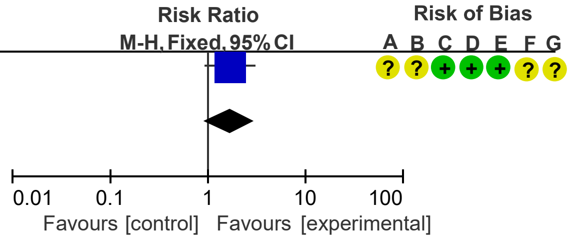

Risk of bias legend
(**A**) Random sequence generation (selection bias)
(**B**) Allocation concealment (selection bias)
(**C**) Blinding of participants and personnel (performance bias)
(**D**) Blinding of outcome assessment (detection bias)
(**E**) Incomplete outcome data (attrition bias)
(**F**) Selective reporting (reporting bias)
(**G**) Other bias

16.3 Disappearance of tenesmus

Study or Subgroup	Experimental Events	Experimental Total	Control Events	Control Total	Weight	Risk Ratio M-H, Fixed, 95% CI	Risk of Bias A	B	C	D	E	F	G
WangT 2012	17	30	9	30	100.0%	1.89 [1.01, 3.55]	?	?	+	+	+	?	?
**Total (95% CI)**		**30**		**30**	**100.0%**	**1.89 [1.01, 3.55]**							
Total events	17		9										
Heterogeneity: Not applicable													
Test for overall effect: Z = 1.98 (P = 0.05)													

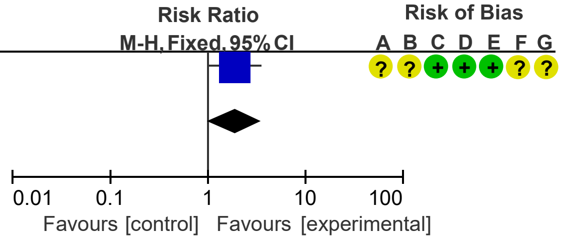

Risk of bias legend
(**A**) Random sequence generation (selection bias)
(**B**) Allocation concealment (selection bias)
(**C**) Blinding of participants and personnel (performance bias)
(**D**) Blinding of outcome assessment (detection bias)
(**E**) Incomplete outcome data (attrition bias)
(**F**) Selective reporting (reporting bias)
(**G**) Other bias

16.4 Recurrence rate

Study or Subgroup	Experimental Events	Experimental Total	Control Events	Control Total	Weight	Risk Ratio M-H, Fixed, 95% CI	A	B	C	D	E	F	G
WangT 2012	1	17	1	9	100.0%	0.53 [0.04, 7.50]	?	?	+	+	+	?	?
**Total (95% CI)**		**17**		**9**	**100.0%**	**0.53 [0.04, 7.50]**							
Total events	1		1										

Heterogeneity: Not applicable
Test for overall effect: Z = 0.47 (P = 0.64)

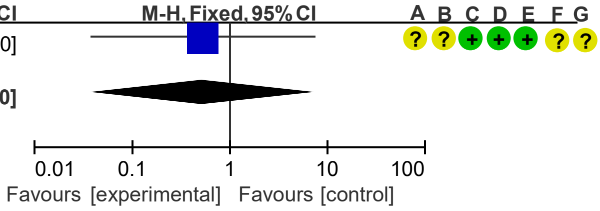

Risk of bias legend
(**A**) Random sequence generation (selection bias)
(**B**) Allocation concealment (selection bias)
(**C**) Blinding of participants and personnel (performance bias)
(**D**) Blinding of outcome assessment (detection bias)
(**E**) Incomplete outcome data (attrition bias)
(**F**) Selective reporting (reporting bias)
(**G**) Other bias

16.5 Disappearance of mucopurulent bloody stool

Study or Subgroup	Experimental Events	Experimental Total	Control Events	Control Total	Weight	Risk Ratio M-H, Fixed, 95% CI	A	B	C	D	E	F	G
WangT 2012	17	30	9	30	100.0%	1.89 [1.01, 3.55]	?	?	+	+	+	?	?
**Total (95% CI)**		**30**		**30**	**100.0%**	**1.89 [1.01, 3.55]**							
Total events	17		9										

Heterogeneity: Not applicable
Test for overall effect: Z = 1.98 (P = 0.05)

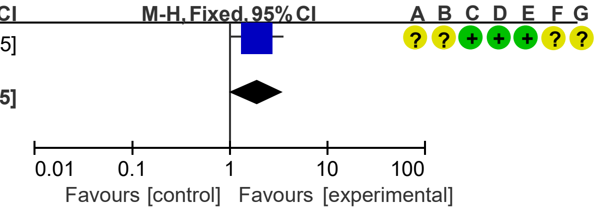

Risk of bias legend
(**A**) Random sequence generation (selection bias)
(**B**) Allocation concealment (selection bias)
(**C**) Blinding of participants and personnel (performance bias)
(**D**) Blinding of outcome assessment (detection bias)
(**E**) Incomplete outcome data (attrition bias)
(**F**) Selective reporting (reporting bias)
(**G**) Other bias

17 SMI+M vs M

17.1 Adverse events

Study or Subgroup	Experimental Events	Experimental Total	Control Events	Control Total	Weight	Risk Ratio M-H, Fixed, 95% CI	A	B	C	D	E	F	G
XuXJ 2008	5	50	6	50	100.0%	0.83 [0.27, 2.55]	?	?	?	?	+	?	?
**Total (95% CI)**		**50**		**50**	**100.0%**	**0.83 [0.27, 2.55]**							
Total events	5		6										
Heterogeneity: Not applicable													
Test for overall effect: Z = 0.32 (P = 0.75)													

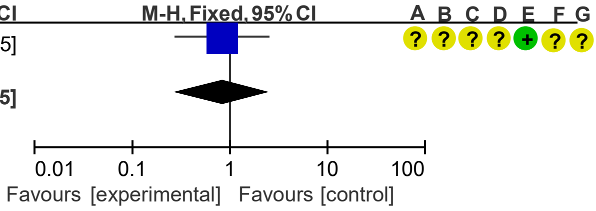

Risk of bias legend
(**A**) Random sequence generation (selection bias)
(**B**) Allocation concealment (selection bias)
(**C**) Blinding of participants and personnel (performance bias)
(**D**) Blinding of outcome assessment (detection bias)
(**E**) Incomplete outcome data (attrition bias)
(**F**) Selective reporting (reporting bias)
(**G**) Other bias

18 ZK+M vs M

18.1 Adverse events

Study or Subgroup	Experimental Events	Experimental Total	Control Events	Control Total	Weight	Risk Ratio M-H, Fixed, 95% CI	A	B	C	D	E	F	G
ChenGH 2007	4	32	3	26	100.0%	1.08 [0.27, 4.41]	+	?	?	?	+	?	?
**Total (95% CI)**		**32**		**26**	**100.0%**	**1.08 [0.27, 4.41]**							
Total events	4		3										
Heterogeneity: Not applicable													
Test for overall effect: Z = 0.11 (P = 0.91)													

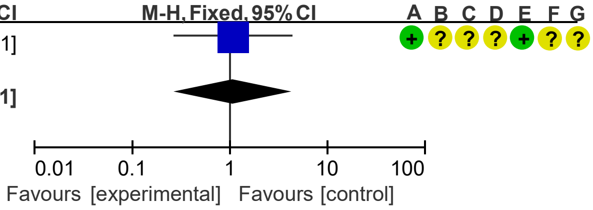

Risk of bias legend
(**A**) Random sequence generation (selection bias)
(**B**) Allocation concealment (selection bias)
(**C**) Blinding of participants and personnel (performance bias)
(**D**) Blinding of outcome assessment (detection bias)
(**E**) Incomplete outcome data (attrition bias)
(**F**) Selective reporting (reporting bias)
(**G**) Other bias

18.2 Disappearance of abdominal pain

Study or Subgroup	Experimental Events	Experimental Total	Control Events	Control Total	Weight	Risk Ratio M-H, Fixed, 95% CI	Risk of Bias A B C D E F G
ChenGH 2007	15	21	8	18	100.0%	1.61 [0.90, 2.88]	+ ? ? ? + ? ?
**Total (95% CI)**		**21**		**18**	**100.0%**	**1.61 [0.90, 2.88]**	
Total events	15		8				
Heterogeneity: Not applicable							
Test for overall effect: Z = 1.59 (P = 0.11)							

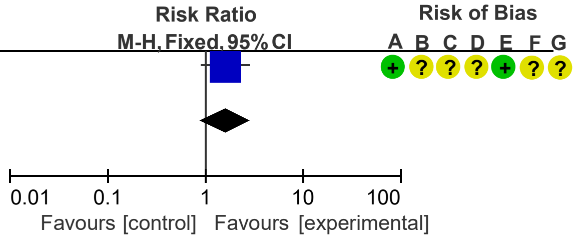

Risk of bias legend
(**A**) Random sequence generation (selection bias)
(**B**) Allocation concealment (selection bias)
(**C**) Blinding of participants and personnel (performance bias)
(**D**) Blinding of outcome assessment (detection bias)
(**E**) Incomplete outcome data (attrition bias)
(**F**) Selective reporting (reporting bias)
(**G**) Other bias

18.3 Disappearance of diarrhea

Study or Subgroup	Experimental Events	Experimental Total	Control Events	Control Total	Weight	Risk Ratio M-H, Fixed, 95% CI	Risk of Bias A B C D E F G
ChenGH 2007	22	30	12	25	100.0%	1.53 [0.96, 2.42]	+ ? ? ? + ? ?
**Total (95% CI)**		**30**		**25**	**100.0%**	**1.53 [0.96, 2.42]**	
Total events	22		12				
Heterogeneity: Not applicable							
Test for overall effect: Z = 1.80 (P = 0.07)							

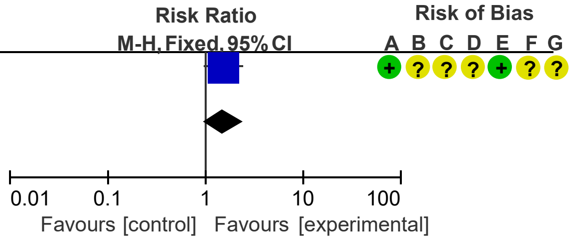

Risk of bias legend
(**A**) Random sequence generation (selection bias)
(**B**) Allocation concealment (selection bias)
(**C**) Blinding of participants and personnel (performance bias)
(**D**) Blinding of outcome assessment (detection bias)
(**E**) Incomplete outcome data (attrition bias)
(**F**) Selective reporting (reporting bias)
(**G**) Other bias

18.4 Disappearance of tenesmus

Study or Subgroup	Experimental Events	Experimental Total	Control Events	Control Total	Weight	Risk Ratio M-H, Fixed, 95% CI	Risk of Bias A	B	C	D	E	F	G
ChenGH 2007	7	24	11	24	100.0%	0.64 [0.30, 1.36]	+	?	?	?	+	?	?
**Total (95% CI)**		**24**		**24**	**100.0%**	**0.64 [0.30, 1.36]**							
Total events	7		11										

Heterogeneity: Not applicable
Test for overall effect: Z = 1.17 (P = 0.24)

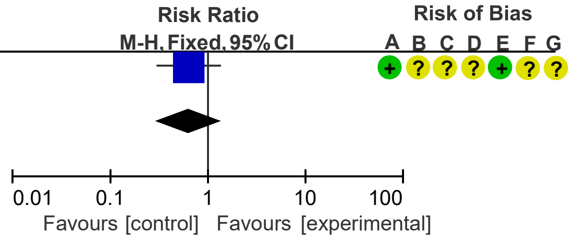

Risk of bias legend
(**A**) Random sequence generation (selection bias)
(**B**) Allocation concealment (selection bias)
(**C**) Blinding of participants and personnel (performance bias)
(**D**) Blinding of outcome assessment (detection bias)
(**E**) Incomplete outcome data (attrition bias)
(**F**) Selective reporting (reporting bias)
(**G**) Other bias

18.5 Disappearance of mucopurulent bloody stool

Study or Subgroup	Experimental Events	Experimental Total	Control Events	Control Total	Weight	Risk Ratio M-H, Fixed, 95% CI	Risk of Bias A	B	C	D	E	F	G
ChenGH 2007	20	26	13	23	100.0%	1.36 [0.90, 2.06]	+	?	?	?	+	?	?
**Total (95% CI)**		**26**		**23**	**100.0%**	**1.36 [0.90, 2.06]**							
Total events	20		13										

Heterogeneity: Not applicable
Test for overall effect: Z = 1.45 (P = 0.15)

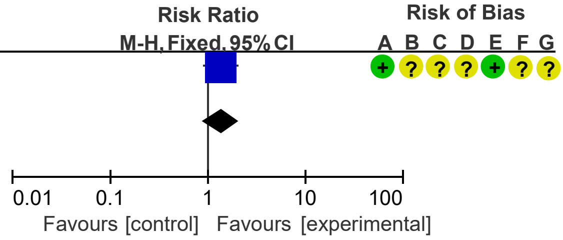

Risk of bias legend
(**A**) Random sequence generation (selection bias)
(**B**) Allocation concealment (selection bias)
(**C**) Blinding of participants and personnel (performance bias)
(**D**) Blinding of outcome assessment (detection bias)
(**E**) Incomplete outcome data (attrition bias)
(**F**) Selective reporting (reporting bias)
(**G**) Other bias

19 FZLZ+M vs M

19.1 Adverse events

Study or Subgroup	Experimental Events	Experimental Total	Control Events	Control Total	Weight	Risk Ratio M-H, Fixed, 95% CI	Risk Ratio M-H, Fixed, 95% CI	Risk of Bias A B C D E F G
LuBY 2019	4	60	3	60	100.0%	1.33 [0.31, 5.70]		? ? ? ? + ? ?
**Total (95% CI)**		**60**		**60**	**100.0%**	**1.33 [0.31, 5.70]**		
Total events	4		3					

Heterogeneity: Not applicable

Test for overall effect: Z = 0.39 (P = 0.70)

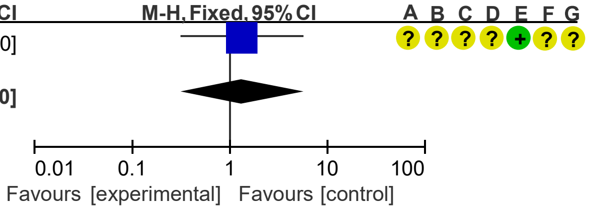

Risk of bias legend

(**A**) Random sequence generation (selection bias)

(**B**) Allocation concealment (selection bias)

(**C**) Blinding of participants and personnel (performance bias)

(**D**) Blinding of outcome assessment (detection bias)

(**E**) Incomplete outcome data (attrition bias)

(**F**) Selective reporting (reporting bias)

(**G**) Other bias

20 BPYC+M vs M

20.1 Adverse events

Study or Subgroup	Experimental Events	Experimental Total	Control Events	Control Total	Weight	Risk Ratio M-H, Fixed, 95% CI	Risk Ratio M-H, Fixed, 95% CI	Risk of Bias A B C D E F G
ZhangC 2012	2	40	6	39	100.0%	0.33 [0.07, 1.51]		? ? ? ? + ? ?
**Total (95% CI)**		**40**		**39**	**100.0%**	**0.33 [0.07, 1.51]**		
Total events	2		6					

Heterogeneity: Not applicable

Test for overall effect: Z = 1.43 (P = 0.15)

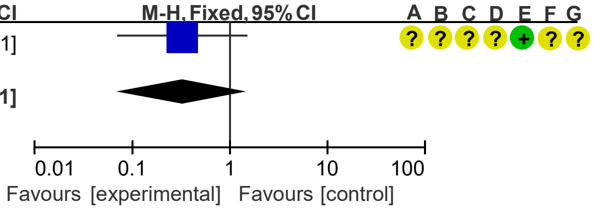

Risk of bias legend

(**A**) Random sequence generation (selection bias)

(**B**) Allocation concealment (selection bias)

(**C**) Blinding of participants and personnel (performance bias)

(**D**) Blinding of outcome assessment (detection bias)

(**E**) Incomplete outcome data (attrition bias)

(**F**) Selective reporting (reporting bias)

(**G**) Other bias

20.2 Disappearance of abdominal pain

Study or Subgroup	Experimental Events	Experimental Total	Control Events	Control Total	Weight	Risk Ratio M-H, Fixed, 95% CI	Risk Ratio M-H, Fixed, 95% CI	Risk of Bias A B C D E F G
ZhangC 2012	38	40	15	39	100.0%	2.47 [1.65, 3.70]		? ? ? ? + ? ?
**Total (95% CI)**		**40**		**39**	**100.0%**	**2.47 [1.65, 3.70]**		
Total events	38		15					

Heterogeneity: Not applicable
Test for overall effect: Z = 4.39 (P < 0.0001)

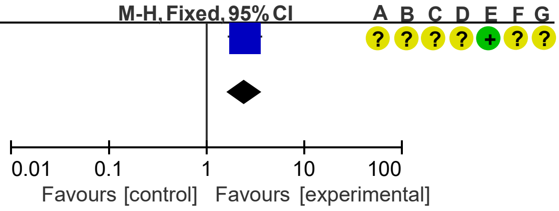

Risk of bias legend
(**A**) Random sequence generation (selection bias)
(**B**) Allocation concealment (selection bias)
(**C**) Blinding of participants and personnel (performance bias)
(**D**) Blinding of outcome assessment (detection bias)
(**E**) Incomplete outcome data (attrition bias)
(**F**) Selective reporting (reporting bias)
(**G**) Other bias

20.3 Disappearance of diarrhea

Study or Subgroup	Experimental Events	Experimental Total	Control Events	Control Total	Weight	Risk Ratio M-H, Fixed, 95% CI	Risk Ratio M-H, Fixed, 95% CI	Risk of Bias A B C D E F G
ZhangC 2012	34	39	18	39	100.0%	1.89 [1.32, 2.71]		? ? ? ? + ? ?
**Total (95% CI)**		**39**		**39**	**100.0%**	**1.89 [1.32, 2.71]**		
Total events	34		18					

Heterogeneity: Not applicable
Test for overall effect: Z = 3.47 (P = 0.0005)

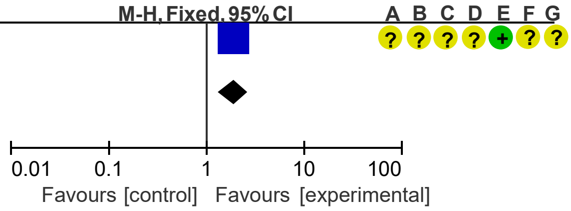

Risk of bias legend
(**A**) Random sequence generation (selection bias)
(**B**) Allocation concealment (selection bias)
(**C**) Blinding of participants and personnel (performance bias)
(**D**) Blinding of outcome assessment (detection bias)
(**E**) Incomplete outcome data (attrition bias)
(**F**) Selective reporting (reporting bias)
(**G**) Other bias
